# A systematic literature review on public health and healthcare resources for pandemic preparedness planning

**DOI:** 10.1186/s12889-024-20629-z

**Published:** 2024-11-11

**Authors:** Berend H. H. Beishuizen, Mart L. Stein, Joeri S. Buis, Alma Tostmann, Caroline Green, Jim Duggan, Máire A. Connolly, Chantal P. Rovers, Aura Timen

**Affiliations:** 1 Centre for Infectious Disease Control, National Institute for Public Health and the Environment, Bilthoven, The Netherlands; 2 Department of Primary and Community Care, Radboud University Medical Centre, Nijmegen, The Netherlands; 3 Department of Medical Microbiology, Radboud Centre for Infectious Diseases, Radboud University Medical Centre, Nijmegen, The Netherlands; 4 School of Computer Science and Insight Centre for Data Analytics, University of Galway, Galway, Ireland; 5 School of Medicine, College of Medicine, Nursing and Health Sciences, University of Galway, Galway, Ireland; 6 Department of Internal Medicine, Radboud Centre for Infectious Diseases, Radboud University Medical Centre, Nijmegen, The Netherlands

**Keywords:** Public health, Systematic review, COVID-19, Bed occupancy, Health Workforce

## Abstract

**Background:**

Generating insights into resource demands during outbreaks is an important aspect of pandemic preparedness. The EU PANDEM-2 project used resource modelling to explore the demand profile for key resources during pandemic scenarios. This review aimed to identify public health and healthcare resources needed to respond to pandemic threats and the ranges of parameter values on the use of these resources for pandemic influenza (including the novel influenza A(H1N1)pdm09 pandemic) and the COVID-19 pandemic, to support modelling activities.

**Methods:**

We conducted a systematic literature review and searched Embase and Medline databases (1995 – June 2023) for articles that included a model, scenario, or simulation of pandemic resources and/or describe resource parameters, for example personal protective equipment (PPE) usage, length of stay (LoS) in intensive care unit (ICU), or vaccine efficacy. Papers with data on resource parameters from all countries were included.

**Results:**

We identified 2754 articles of which 147 were included in the final review. Forty-six different resource parameters with values related to non-ICU beds (*n* = 43 articles), ICU beds (*n* = 57), mechanical ventilation (*n* = 39), healthcare workers (*n* = 12), pharmaceuticals (*n* = 21), PPE (*n* = 8), vaccines (*n* = 26), and testing and tracing (*n* = 19). Differences between resource types related to pandemic influenza and COVID-19 were observed, for example on mechanical ventilation (mostly for COVID-19) and testing & tracing (all for COVID-19).

**Conclusion:**

This review provides an overview of public health and healthcare resources with associated parameters in the context of pandemic influenza and the COVID-19 pandemic. Providing insight into the ranges of plausible parameter values on the use of public health and healthcare resources improves the accuracy of results of modelling different scenarios, and thus decision-making by policy makers and hospital planners. This review also highlights a scarcity of published data on important public health resources.

**Supplementary Information:**

The online version contains supplementary material available at 10.1186/s12889-024-20629-z.

## Introduction

In the 21st century, two pandemics caused by respiratory viruses of zoonotic origin have been declared by the World Health Organization (WHO): the novel influenza A(H1N1)pdm09 pandemic and the COVID-19 pandemic. The impact of the COVID-19 pandemic was unprecedented, as it involved all public health and healthcare domains and revealed important weaknesses of the health system. At various stages of the COVID-19 pandemic, health systems were unable to cope with the demand, for example on testing and tracing [1], hospital capacity [2, 3], stocks of personal protective equipment (PPE) [4] and morgue capacity [5].

Mathematical modelling is a key element for infectious diseases control, and its use has become more widespread in recent decades due to the increased availability of electronic data and computing power [6]. During the COVID-19 pandemic, models for epidemiological analysis and forecasting have been used by public health institutes and governments to inform policy, and also by universities and members of the public using publicly available epidemiological data. However, these epidemiological models generally did not provide direct insights in public health and healthcare resource demands [7]. Models that link projections of epidemiological outcomes with public health and healthcare resource capacities can be applied during multiple stages of the preparedness cycle [8]: to inform scenario development for governance, capacity building and maintenance, and risk assessment during the pre-event or cold phase, and for short-term forecasting to support risk and crisis management during the event or hot phase of an outbreak [9, 10]. Resource demands differ greatly between, and also within prolonged outbreaks and pandemics due to different epidemiological progression, availability (or lack thereof) and effectiveness of treatments or vaccines and different application of non-pharmaceutical interventions. This makes data on the availability and use of resources (pharmaceutical and non-pharmaceutical) extremely valuable, Addressing the resource modelling gap was one of the aims of PANDEM-2, an EU Horizon2020 project [11], of which this review was a part. PANDEM-2 delivered a dashboard with tools for pandemic preparedness and response, including a robust mathematical model that can inform policy makers on resource demands and utilisation, which was piloted during a functional exercise [12].

Modelling relies on robust, preferably real-world data to deliver accurate outcomes. Epidemiological data are widely available and have also been systematically collected for modelling purposes [13]. This is, however, not the case for data on public health and healthcare resources utilization, which are usually not systematically collected in a standardized and centralized manner, nor are they part of routine infectious disease surveillance systems. This review sought to answer the question which public health and healthcare resource data from recent pandemics is available in scientific literature. In the context of this review, we define public health and healthcare resources as those directly relevant to the control or mitigation of a pandemic from a public health and healthcare perspective, as they are most relevant input data for modelling for pandemic preparedness or forecasting during pandemics. Non-medical resource costs, costs of non-pharmaceutical interventions and indirect costs (such as productivity loss) are of course extremely important for policy decisions, but they are beyond the scope of this work. The aim of this review therefore was to identify what resource data is available from scientific research on recent pandemics (the novel influenza A(H1N1)pdm09 and COVID-19 pandemics), and provide a comprehensive overview of resource data useful to mathematical modellers.

## Methods

### Scope

We provide an overview of public health and healthcare resources most likely to be needed in pandemics of respiratory pathogens, comparable to the novel influenza A(H1N1)pdm09 pandemic and the COVID-19 pandemic. Historic pandemics of a similar nature are not included as the advances in healthcare systems and medical countermeasures, including vaccines and technology, mean that their data may no longer be applicable to pandemic management in the 21st century.

### Methodology

The systematic literature review was conducted according to guidelines presented in the PRISMA statement (Supplementary material 1) [14] and used the PCC (population, concept, context) framework to develop our research question and inform the search strategy, as it is more suitable for explorative review than the PICO (population, intervention, comparators, outcomes) framework commonly used for systematic literature reviews and meta-analysis [15]. Embase and Medline databases were searched through Embase.com using a search strategy developed with assistance from a scientific information specialist (Supplementary material 2). The search was first performed on the 21st of May 2021 and updated on the 25th of March 2022 and again on the 3rd of June 2023. Endnote 20 software [16] was used to support the review process.

### Study selection

We included articles that (i) contain a model, scenario or simulation related to pandemic resources citing resource parameter data, or (ii) observational studies describing resource parameters and associated values or rates. No specific population was targeted due to the diverse nature of studies we expected to include resource data from. We included articles published in the context of pandemics with a respiratory pathogen (pandemic influenza, novel influenza A(H1N1)pdm09 or COVID-19). We excluded articles written in a language other than English or Dutch (the native language of the researchers), articles on seasonal influenza, articles with non-human data, articles on historic pandemics of respiratory pathogens (such as the 1918–1920 flu pandemic) and articles published before 1995. The reference lists of review articles identified by our search strategy were screened for relevant original research papers. Commentaries, conference abstracts or editorials were excluded.

Articles were assessed by two researchers independently by screening the title and abstract, and subsequently by screening full texts of articles included after the first step. Disagreements about the inclusion of an article were resolved via discussion. If both researchers were still unsure about inclusion or rejection, a third researcher was consulted.

### Data extraction

The following data were extracted from included articles: first author, year published, country and World Bank classification [17], study period, study design, study setting and disease type In relation to resource data, we extracted the resource type and specific parameter, and the associated value.

### Study quality

The aim of this review is to identify and extract resource parameter data from scientific publications for the purposes of mathematical modelling, and not to perform a meta-analysis of found resource parameters. Therefore, the 147 studies selected were not assessed for risk of bias within the individual studies or across studies, and no quality assessment was performed prior to data extraction.

## Results

### Search results

**Fig. 1:**
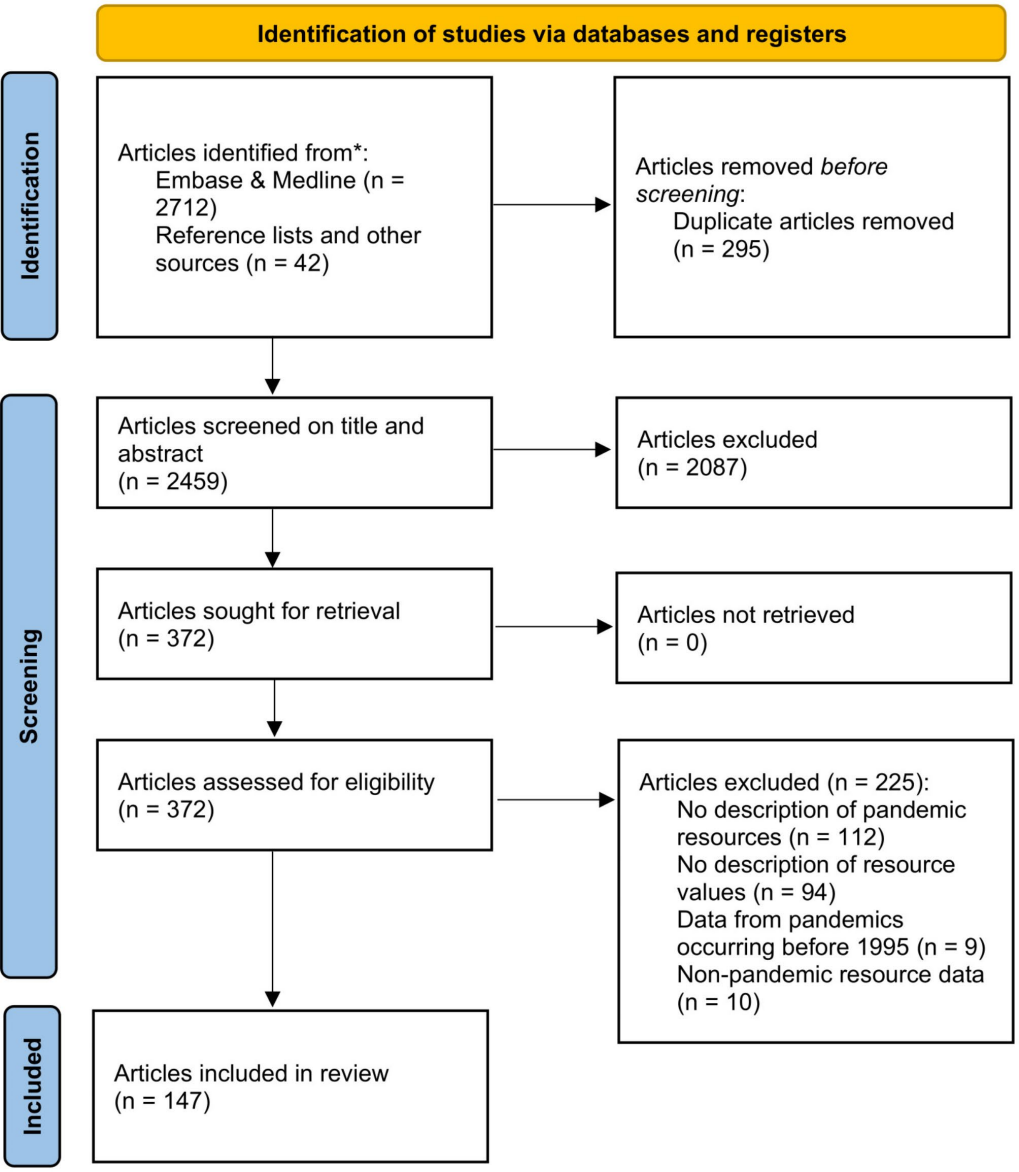
PRISMA flowchart of studies included in this review

The initial search yielded 1166 articles and the two repeated searches added a further 788 and 758 articles to a total of 2712. After removing duplicates (8, 211, and 76 for the first, second and third searches respectively), the combined search yielded 2417 articles. Additionally, 40 articles were identified from reference lists and previous work done in the AsiaFluCap project [18]. In total, 2457 articles were screened for inclusion based on title and abstract (Fig. 1). 2087 articles were excluded, leaving 370 articles for full text analysis. Following full text analysis, 225 articles were excluded because they either did not contain a description of pandemic resources (*n* = 112), had a description of resources but did not report resource values (*n* = 94), described pandemics occurring before 1995 (*n* = 9) or reported resource data in settings unrelated to a pandemic (*n* = 10). Following our screening process, 145 articles were finally included in this systematic review.

### Study characteristics

We included 45 (31%) studies related to pandemic influenza, including novel influenza A(H1N1)pdm09, and 102 (69%) related to the COVID-19 pandemic (Table 1). Studies were conducted in 53 different countries, with the majority conducted in Europe (38%), North America (32%) and.

**Table 1:** Summary of study characteristics for the articles included in this review

	Total (*n* = 147)	Pandemic influenza^a^ (*n* = 45)	COVID-19 (*n* = 102)
**Region of study**^b^** (***n*, **%)**			
Africa	5 (3.3%)	0 (0%)	5 (4.9%)
Asia	31 (20.7%)	11 (23.4%)	20 (19.4%)
Europe	57 (38%)	13 (27.7%)	44 (42.7%)
North America	48 (32%)	18 (38.3%)	30 (29.1%)
Oceania	5 (3.3%)	5 (10.6%)	0 (0%)
South America	4 (2.7%)	0 (0%)	4 (3.9%)
**World Bank classification**^**c**^ [17] **(***n*,** %)**			
High income	119 (82.3%)	42 (91.3%)	77 (78.2%)
Upper middle income	15 (10.2%)	3 (6.5%)	12 (11.9%)
Lower middle income	11 (7.5%)	1 (2.2%)	10 (9.9%)
**Study approach (***n*,** %)**			
Clinical trial	3 (3.4%)	1 (2.2%)	2 (3.9%)
Cost-benefit analysis	1 (0.7%)	1 (2.2%)	0 (0%)
Evaluation	19 (12.9%)	6 (13.3%)	13 (12.7%)
Model building	70 (47.6%)	34 (75.6%)	36 (35.3%)
Observational	50 (34%)	2 (4.4%)	48 (47.1%)
Survey	2 (1.4%)	1 (2.2%)	1 (1%)
**Research focus**^**d**^**(***n*,** %)**			
Contact tracing	3 (2%)	0 (0%)	3 (2.9%)
First responder^e^	3 (2%)	0 (0%)	3 (2.9%)
General practice	1 (0.7%)	0 (0%)	1 (1%)
Hospital	102 (70.3%)	33 (73.3%)	69 (68.9%)
Long-term care facilities	1 (0.7%)	0 (0%)	1 (1%)
Emergency preparedness	8 (5.4%)	4 (8.9%)	4 (3.9%)
Public health	8 (5.4%)	3 (6.7%)	5 (4.9%)
School	1 (0.7%)	1 (2.2%)	0 (0%)
Testing strategies	6 (4.1%)	0 (0%)	6 (5.8%)
Vaccination	13 (8.8%)	4 (8.9%)	9 (8.7%)

^a^Including novel influenza A(H1N1)pdm09

^b^Three studies included countries from multiple regions: 2x North America & Europe, 1x North America, Europe & Asia. One study includes global data and is not included in this count

^c^One study includes global data and is not included in this count. One study includes data from both upper middle income and lower middle income countries

^d^One study includes data on both contact tracing and testing strategies

^e^Includes paramedics and fire fighters responding to emergencies

Asia (20.7%). Several studies (*n* = 11, 7.5%) were conducted in multiple countries. Most studies were conducted in high-income countries (*n* = 119; 82.3%), followed by upper-middle income (*n* = 15; 10.2%) and lower-middle income (*n* = 11; 7.5%). Most included studies were model building studies (*n* = 70; 47.6%), followed by observational studies (*n* = 50; 34%), evaluations (*n* = 19; 12.9%), clinical trials (*n* = 5; 3.4%), surveys (*n* = 2; 1.4%) and cost-benefit analyses (*n* = 1; 0.7%). The majority of studies relating to pandemic influenza were model building studies, while for COVID-19 and the majority were observational studies, followed by model building studies. Research focus was similar for pandemic influenza and COVID-19, with the majority being hospital related. Studies relating to COVID-19 had slightly more diverse foci than those on pandemic influenza (9 vs. 5 different foci). The full list of included studies and characteristics is given in Supplementary Table 1.

### Pandemic resources identified from literature

We identified 15 different resources with a total of 46 different resource parameters related to pandemic influenza and COVID-19 pandemic (Table 2). Resources were identified for both pandemic influenza and COVID-19, or either only for pandemic influenza or only for COVID-19.

**Table 2:** Overview of resources and associated parameters identified in this systematic review. ICU: Intensive Care Unit; ECMO: Extra Corporeal membrane oxygenation; HFNO: high Flow Nasal Oxygen; CPAP: continuous positive Airway pressure; PPE: personal protective equipment

Healthcare resources		Public health resources	
Non-ICU beds - Admission rate - Length of stay - Readmission rate ICU Beds - Admission rate - Length of stay - Surge capacity Readmission rate - Mechanical ventilation - Use rate - Duration - ECMO use - HFNO use - CPAP use Healthcare workers - Absenteeism - HCW to bed/patient ratio Antibiotics - Dosage	Antivirals - Dosage - Efficacy - Stockpiling - Use rate Other pharmaceuticals - Dosage - Use rate PPE - Use rate - Stockpiling - Surge demand Ambulances - Arrival time General practitioners - Consultation rate Palliative care - Care kits - Use rate - Duration Care seeking - Outpatient care - Inpatient care	Vaccines - Dose frequency - Vaccine administration speed - Vaccination rate - Manufacturing capacity - Efficacy - Effectiveness against transmission - Breakthrough transmission	Contact tracing - Adherence - Success rate - Speed - Testing - Laboratory processing time - Time to test result Testing capacity - Test sensitivity - Test specificity

### Healthcare resources

#### Non intensive care unit beds

Use of general clinical departments (i.e. non-intensive care unit; non-ICU beds or ward beds) was reported in 43 publications: 14 for pandemic influenza and 29 for COVID-19 (Table 3). Usage was defined by the parameters admission rate and length of stay (LoS). For pandemic influenza, the non-ICU admission rate ranged between 0.4 and 11% for symptomatic cases [19–24], and 3% admission rate for patients who consulted a general practitioner (GP) [25], with an average LoS of 5–11 days [26–32]. For COVID-19, reported non-ICU admission rates were between 1 and 37.6% for all symptomatic cases [33–39], 8.4% of all laboratory confirmed covid cases [40],466.7 per 100,000 adults [41], 73.1% of confirmed COVID-19 cases in the emergency department [42] or 17.4% of cases from outbreaks in long-term care facilities [43]. One study reported on admission rates for non-vaccinated (886 per 100,000), vaccinated (92 per 100,000) and boosted (43 per 100,000) individuals aged ≥ 12 years [44]. One study reported on admission rates per COVID-19 wave: pre-wave 1 (March – June 2020): 22.3%, wave 1 (June – August 2020): 16.8%, post-wave 1 (August – November 2020): 22.2%, wave 2 (November 2020 – February 2021): 19.6% of all covid cases [45]. The average reported non-ICU LoS for COVID-19 patients varied between 2 and 31.8 days [20, 34–37, 39, 45–57].One study specifically reported on pre- and post-ICU LoS on general wards: 2.7 and 13.6 days respectively [58]. One study reported a non-ICU LoS of 15.6 days during the first wave and a LoS of 11.6 days during the second wave [59]. One study reported on a non-ICU LoS of 9.2 days pre-Delta, 9.7 days during Delta and 7.1 days during Omicron; and non-ICU LoS of 10.6 days for vaccinated patients during Delta and 6.8 days LoS for vaccinated patients during Omicron, and 9.4 days for non-vaccinated patients during Delta and 7.8 days for non-vaccinated patients during Omicron [60].

**Table 3:** Description of extracted resource parameters for non-intensive care unit (ICU) beds, ICU beds, mechanical ventilation, healthcare workers and vaccines. Data stratified by different COVID-19 waves or vaccination status are not included in this table. GP: general practitioner, ED: emergency department, ECMO: extra corporeal membrane oxygenation, HFNO: high flow nasal oxygen, CPAP: continuous positive airway pressure, VET: vaccine effectiveness against transmission

	Pandemic influenza^a^	COVID-19
**Non-ICU beds**		
Number of studies	14	29
Admission rate		
(% of symptomatic cases)	0.4–11%	-
(% of all cases)	-	1-37.6%
(% of GP consultations)	3% of patients who consulted a GP	-
(% of confirmed cases visiting ED)	-	73.1%
(% of laboratory confirmed cases)	-	8.4%
(per 100000 adults)	-	466.7
Length of stay (days)	5–11	2-31.8
**ICU beds**		
Number of studies	16	41
Admission rate		
(% of hospital admissions)	12.8–36%	10.6–66%
(% of all cases)	-	4.6–8.7%
(% of COVID-19 cases visiting ED)	-	13.4%
(% of all laboratory confirmed cases)	-	1.4%
(per 100000 adults)	-	136.6
Length of stay (days)	5.8–12	3-26.9
Surge capacity (%)	-	115–1111%
**Mechanical ventilation**		
Number of studies	10	29
Need for mechanical ventilation		
(% of hospitalized patients)	7.5–30%	7-39.8%
(% of ICU patients)	50-93.2%	26.4–88%
(% of all COVID-19 confirmed cases)	-	6.1%
Duration (days)	7–14	8–18
ECMO use		
(% of all COVID-19 hospitalizations)	-	0.2–1.5%
(% of ICU patients)	-	3–31%
HFNO use		
(% of all COVID-19 hospitalizations)	-	0.5–3.3%
(% of ICU patients)	-	5-17.4%
CPAP use		
(% of all COVID-19 hospitalizations)	-	0.2%
(% of ICU patients)	-	48.6%
**Healthcare workers**		
Number of studies	5	7
Absenteeism (prevalence %)	10-56.6%	3.3–17%
Capacity	5 patients per regular ward nurse 10 patients per regular ward physician	0.4–1.6 patients per ICU nurse 7 patients per intensivist
**Vaccines**		
Number of studies	15	11
Dose frequency	1–2 per patient	-
Efficacy (%)	35–52% for susceptibility	Symptomatic COVID-19: 35% (1 dose BNT162b2), 59% (2 doses) Asymptomatic COVID-19: 35% (1 dose BNT126b2), 49% (2 doses)
	80% for illness given infection	94.6–95 (BNT162b2) 93.6 (Moderna) 60 (AstraZeneca) 65.8–67 (Janssen, single dose)
Efficacy, other	Vaccinated elderly patients: 1.31 chest X-rays Unvaccinated elderly patients: 2.61 chest X-rays	VET of BNT162b2: 96% (Alpha), 87% (Delta), 31% (Omicron). VET of BNT162b2 booster: 87% (Delta), 68% (Omicron)
	Antibiotics duration vaccinated patients: 2.32 days (oral), 2.55 days (IV) Antibiotics duration unvaccinated patients: 3.98 days (oral), 7.52 days (IV)	-
Administering speed	17.5–30 vaccines per nurse per hour	1284–50,000 per day 0.27–8.27 doses per 100 people per day
Manufacturing capacity	22 million doses per week (globally)	-

^a^Including novel influenza A(H1N1)pdm09

#### Intensive care unit beds

Data on ICU bed use was reported in 57 studies (16 for pandemic influenza, 41 for COVID-19) (Table 3). Similar to the data on non-ICU beds, admission rate and LoS were identified as resource parameters. Additionally for COVID-19,surge capacity was also identified as an ICU bed parameter. For pandemic influenza, admission rates were all reported as a percentage of all hospitalized patients (12.8–36%) [21, 23, 24, 26, 29, 30, 61–64]. The average LoS on the ICU was 5.8–12 days [25, 26, 28–30, 46, 63, 65–67]. For COVID-19, ICU admission rates were either reported as percentage of all COVID-19 cases (4.6-8.7%) [35, 36, 68, 69], percentage of all COVID-19 hospitalizations (10.6–66%) [20, 33, 34, 37, 39, 44, 46, 47, 57, 70–76], percentage of COVID-19 cases presented at emergency department (ED) (13.4%) [42], as percentage of all laboratory confirmed cases (1.4%) [40] or as 136.6 per 100,000 adults [41]. One study reported admission rates stratified by vaccination status (230.6 per 100,000 for non-vaccinated individuals, 30.8 per 100,000 for vaccinated individuals and 5.5 per 100,000 for boosted individuals) [77]. One study on ICU admission rates as 28.7% of hospitalized patients pre-Delta, 21.4% during Delta and 15.4% during Omicron, and ICU admission rates of vaccinated hospitalized patients as 22.8% during Delta and 14.2% during Omicron, and for non-vaccinated hospitalized patients 19.3% during Delta and 14.7% during Omicron [60]. Average LoS was reported as 3-26.9 days [20, 34–37, 39, 49, 51–53, 55–59, 69, 70, 75, 78–81]. One study reported on ICU LoS of 9.8 days pre-Delta, 9.4 days during Delta and 6.3 days during Omicron; and ICU LoS of 8 days for vaccinated patients during Delta and 4.8 days LoS for vaccinated patients during Omicron, and 11.6 days for non-vaccinated patients during Delta and 5.9 days for non-vaccinated patients during Omicron [60]. Surge capacity was reported as the possible percentage of normal non-pandemic ICU beds (115–1111%) [74, 82–86].

#### Mechanical ventilation

Mechanical ventilation was reported in 39 studies, ten relating to pandemic influenza and 29 for COVID-19 (Table 3). For pandemic influenza, mechanical ventilation was mainly reported as the percentage of patients requiring mechanical ventilation in ICU (50-93.2%) [21, 23, 26, 66] or in all hospitalized patients (7.5–30%) [61, 87–89]. The type of mechanical ventilation was not specified. One study reported on the duration of mechanical ventilation for ICU patients with pandemic influenza (60% of patients require 1 week, 40% require two weeks) [21]. For COVID-19, we identified rates of need for mechanical ventilation, type unspecified (26 studies), extracorporeal membrane oxygenation (ECMO, six studies, 0.2–1.5% of all COVID-19 hospitalizations [55, 60, 75] and 3–31% of ICU patients [54, 80, 90]), high-flow nasal oxygen (HFNO, four studies, 0.5–3.3% of COVID-19 hospitalizations [59, 71] and 5-17.4% of ICU patients [67, 90]) and continuous positive airway pressure (CPAP, two studies, 0.2% of all COVID-19 hospitalizations [59] and 48.6% of ICU patients [67]). The reported rates of use for unspecified mechanical ventilation were 6.1% for all COVID-19 cases [35], 7-39.8% of COVID-19 hospitalizations [48, 53, 56, 59, 68, 71, 72, 75, 76, 91, 92] and 26.4–88% for ICU patients [20, 24, 33, 37, 49, 54, 55, 69, 74, 93–95]. One study reported on mechanical ventilation use of 11.8% for all COVID-19 hospitalizations pre-Delta, 13.4% during Delta and 7.6% during Omicron, and mechanical ventilation use of 12.4% for all vaccinated COVID-19 hospitalizations during Delta and 6.4% during Omicron, and 13.5% for all non-vaccinated COVID-19 hospitalizations during Delta and 7.2% during Omicron [60]. Eight studies also reported on the duration of mechanical ventilation for COVID-19 patients (median 8–18 days) [20, 35, 49, 54–56, 78, 90].

#### Healthcare workers

Parameters concerning healthcare workers (HCW) were identified in five studies for pandemic influenza and seven studies for COVID-19 (Table 3). For general HCW, absenteeism during the H1N1 influenza pandemic was reported as between 10 and 30% [87, 89, 96]. For emergency department staff, one study reported 36% becoming ill with influenza-like illness, and of these, 56.6% having at least one day of absence and a mean of 3.73 days of absence [97]. For COVID-19, absenteeism due to illness was reported as 3.3–14.6% for HCWs [93], 12.1% for ICU nurses [98] and 17% for general practice frontline workers [99]. Absenteeism due to burnout (6.9%) was also reported for HCWs during the COVID-19 pandemic [3]. Healthcare worker capacity was reported as the number of patients that can be attended per nurse or physician per shift, or number of nurses per bed. For pandemic influenza, this was reported as 10 patients for one physician and five patients for one nurse during daytime shifts (40 per physician and 20 per nurse during night time shifts) [100]. For COVID-19, this was reported for intensive care unit (ICU) capacity as 1-1.6 patients per nurse per shift [101, 102] or 2.26:1 nurse to bed ratio [74], seven patients per intensivist and 4 patients per respiratory therapist [101].

#### Antivirals, antibiotics and other pharmaceuticals

Twenty-one studies reporting on antivirals, antibiotics or other pharmaceuticals as a resource for treatment were identified, 10 regarding pandemic influenza and 11 regarding COVID-19 (Supplementary Table 2). For pandemic influenza we found oseltamivir prophylaxis or treatment (one dose of 75 mg per day for prophylaxis, two doses per day for 5–7 days for treatment) [65, 100, 103–105] and efficacy (60–89%) [106–108], zanamivir treatment (20 mg per day for 5 days) [103] and efficacy (61–62%) [106, 107], amantadine treatment (200 mg daily) [103] and efficacy (61%) [106] and rimantadine efficacy (72%) [106]. Two studies also reported on the required or estimated stockpile of antivirals (recommending a stockpile sufficient for either 4–6% [19] or 25% [109] of the population). One study also reported on the use of antibiotics (amoxicillin and co-trimoxazole) in patients with pandemic influenza [100].

For COVID-19, studies reported remdesivir treatment (200 mg on day 1, followed by 100 mg per day up to day 10) [110, 111], rate of use (4.5% of ICU patients [67] or 2.8% of all COVID-19 hospitalizations [112]) and effect (30% relative reduction in progression to ICU [39]). One study reported on general use of antivirals during the first wave (6.7% of all COVID-19 hospitalizations) and the second wave (1.5%) [59]. Dexamethasone was also reported as a treatment for COVID-19, with treatment (1 dose of 6 mg per day up to 10 days) [110, 113] and rate of use (2.2% of COVID-19 patients in March-June 2020 [71], 34% of COVID-19 hospitalizations in January 2022 [114]). Use of steroids as therapeutics was reported in two publications as 9.5% of all COVID-19 hospitalizations during the first wave and 28% during the second [59] and 11.1% of all COVID-19 hospitalizations during the second wave [112]. Two studies on the effect of hydroxychloroquine were included. One study reported a dosage of 600 mg per day and no reduction in ICU admissions or deaths in hospitalized COVID-19 pneumonia patients receiving oxygen, and no effect on survival in hospitalized COVID-19 patients without acute respiratory distress syndrome [115]. The other study reported a range of dosages (200 mg, 400 mg and 600 mg per dose, either once or twice daily) and no significant differences in in-hospital mortality between patients receiving hydroxychloroquine treatment and control group [116].

#### Personal protective equipment

Eight studies reported on the usage of personal protective equipment (PPE), with six regarding pandemic influenza and two regarding COVID-19 (Supplementary Table 2). For pandemic influenza, three studies reported on the composition of PPE kits in hospitals [89, 117, 118] and two on the rate of use: 20–25 PPE kits per patient in the first six hours of treatment [119], or 91 surgical masks and 40 N95 masks per hospitalized patient [100]. One study reported on stockpiling PPE kits for pandemic influenza for a hospital [117]. For COVID-19, we found a reported daily use of 195 PPE kits per occupied hospital bed during March & April 2020 [120], and an increased demand for surgical masks (355%), surgical gowns (1105%), plastic aprons (443%) and hand sanitizer (136%), also during the first wave of COVID-19 [121].

#### Other healthcare resources

Several publications reporting on other aspects of care as pandemic related resources were identified (Supplementary Table 2). Three studies reported on delayed arrival times (5–15 min) [122–124] for ambulances during the COVID-19 pandemic. Two studies reported on GP consultations related to pandemic influenza: 12% of the population consulted the GP [25], one patient visited the GP an average of 2.1 times [63] and 60.5% of hospitalized patients have visited the GP before or after hospitalization for pandemic influenza [63]. One study reported on care seeking behaviour during the COVID-19 pandemic: 83% of those tested who positive sought outpatient care during the 1st wave, 81% (2nd wave), 69% (3rd wave) and 59% (4th wave); for inpatient care this amounted to 4.9% of those who tested positive during the 1st wave, 3.6% (2nd wave), 1.4% (3rd wave) and 0.5% (4th wave) [125]. One study reported that COVID-19-related readmissions within 30 days occurred for 5.3% of all COVID-19 hospitalizations, and readmission to ICU for 1.3% of COVID-19 hospitalizations; for 4.1% of COVID-19 outpatients COVID-19-related hospitalization occurred within 30 days, for 0.7% this was COVID-19-related ICU admission [75]. For palliative care during the COVID-19 pandemic, one study reported the need for palliative care for 22.3% of hospitalized patients, with an average duration of 2 days [126]. One study made recommendations on the need for stockpiling of, and contents of palliative care kits [127].

### Public health resources

#### Vaccines

We identified 15 studies reporting on vaccine data for pandemic influenza, and 11 studies for COVID-19 (Table 2). On vaccine dose frequency for pandemic influenza, five studies reported using two doses to fully vaccinate a person [104, 108, 128–130], while two studies from the United States reported one dose with maximum efficacy obtained after two weeks [28, 131]. Vaccination rate, defined as the number of vaccines administered per hour or persons vaccinated per hour, was reported in two studies: one at the rate of 17.5 vaccines per vaccinator per hour [128], based on an exercise carried out by a public health service, and the other at the rate of 30 per hour [132], based on guidelines set by the US Centres for Disease Control and Prevention (CDC). Manufacturing capacity for vaccines was reported on in two ways: first as the time it takes to develop a new vaccine (at least 6 months [133]) and second as capacity in doses per week (22 million monovalent doses globally, egg-based vaccine technology in 2004 [129]). Vaccine efficacy was also reported on in various ways for pandemic influenza: as reduction in chest X-rays (50%) and duration of antibiotic treatment (58% shorter) [134], as susceptibility (59–87%) [24, 135–137], as reduction of infectiousness (35–40%) [131, 136] and as reduction of illness given infection (80%) [131]. Two studies also reported on vaccine stockpiles for pandemic influenza (stockpiling for 30% of the population [132] with expected shelf-life of 4 years [138]). For COVID-19, five studies reported on vaccine efficacy. One study reported effectiveness of two doses BNT162b2 against of asymptomatic (0.49) or symptomatic infection (0.59) with the delta variant [139]. Data on vaccine efficacy of different vaccine types were identified as 94.6–95% for Pfizer/BioNTech [140, 141], 93.6% for Moderna [140], 60% for Moderna [140] and 65.8–67% for Janssen, single dose [140–142]. One study reports on the vaccine effectiveness of various combinations and doses against the delta variant: two doses CoronaVac and one dose BNT162b2: 98%, two doses CoronaVac and one dose ChAdOx1 nCoV-19: 86%, two doses ChAdOx1 nCoV-19: 83%, one dose CoronaVac + one dose ChAdOx1 nCoV-19: 74%, two doses CoronaVac: 60%; one dose of either CoronaVac or ChAdOx1 nCoV-19: <50% [143]. One study reported on vaccine effectiveness against transmission (VET) of primary BNT162b2 vaccination of 96% against the alpha variant, 87% against delta and 31% against omicron; and VET of booster vaccination was 87% against delta and 68% against omicron, decreasing to 71% (delta) and 55% (omicron) 150–200 days after booster vaccination [144]. One study also reports a breakthrough transmission rate of 10.1% from fully vaccinated patients to close contacts compared to 37.8% breakthrough transmission in the control group of unvaccinated patients to close contacts [145]. Five studies report on vaccination rates for COVID-19 during rollout of the vaccination campaigns in 2021. One study reports 0.27 doses per 100 people in Oman, 0.39 doses per 100 in Saudi Arabia, 3.92 doses per 100 people in Bahrain and 8.27 doses per 100 people in the United Arab Emirates [146]. Four studies report on the total amount of vaccinations administered per day: 4024 per day in the Basque Country, Spain [140]; up to 50,000 per day in Cambodia [147]; 1284 per day in Djibouti [148] and 42,249 per day in South Korea [149].

#### Testing and contact tracing

Studies on testing and contact tracing of people as a resource were only identified for COVID-19, totalling 19 studies, 16 on testing and three on contact tracing (Supplementary Table 2). Resource parameters for testing were processing time in laboratories (5 h for polymerase chain reaction (PCR) tests) [150] and time to test result (< 24 h from sample taking to delivery of test result for PCR or antigen tests [151, 152]) and test sensitivity (0.95–0.99 for PCR [150, 152–154] and 0.50–0.80 for lateral flow antigen (LFA) tests [152, 153, 155, 156]) and specificity (0.99 for PCR [154], 0.98–0.99 for LFA tests [152, 155, 156]). One study also reported on sensitivity of LFA tests in specific situations: 55.7% for the alpha variant, 64% for delta and 73% for omicron; 57.3% in case of unvaccinated individuals, 67.6% for those with one dose and 69.7% for those with two or more vaccine doses; and 68.7% in symptomatic cases vs. 52.8% in asymptomatic cases [156]. Amounts of tests performed were reported either as total amounts per 100,000 population (17.6–217 [150, 157, 158]), or number of tests per day, for PCR testing (137-2000000 per day, settings ranging from a mobile container testing facility to the United States [40, 85, 147, 148, 151, 152, 159–161]) or LFA testing (28-3002 per day, settings ranging from a mobile container testing facility to the Veteran’s Health Administration [40, 151, 161]).

For contact tracing, adherence to contact tracing measures (18.2% full adherence to isolation) [153] was reported in one study. Two studies reported on the success rate of contact tracing, reporting either success of reaching contacts (99.5% within same day for household contacts, 85% within two days for school place contacts, 60% within two days for workplace contacts, 10% within three days for community contacts [162]) or other aspects of contact tracing (cases interviewed: 49%, interviewed cases who named contacts: 25%, contacts who were notified: 59%, contacts who were monitored: 32% [163]). This last study also reported on the speed of contact tracing, with it taking 3.5 days from testing to a case interview, and 4 days from testing to contact notification [163].

## Discussion

This systematic review identified public health and healthcare resources with their associated parameters and values reported in relation to the pandemic influenza and COVID-19 pandemics. We found 147 studies, 45 related to pandemic influenza and 102 related to COVID-19, and identified 16 resource types and 48 resource parameters. As nearly 70% of the studies included were performed in hospitals, we found more resources related to healthcare than to public health. The data identified in this review will be of great benefit to resource modelling for preparedness and outbreak management because relevant model outputs rely on realistic input data. For preparedness activities, this will allow pandemic planners and policy makers to improve estimations for resource needs and stockpiles in preparedness plans and support simulation-based training. For outbreak or pandemic management, more accurate short-term forecasting will better inform decision making for outbreak management policy and containment measures, both at public health and clinical level.

Most studies identified were on ICU and non-ICU bed-related resources, followed by mechanical ventilation and vaccines. All studies on testing and tracing were in relation to COVID-19, as testing and tracing were cornerstones of the measures to control COVID-19 and contact tracing was mainly used only in the initial containment phase of the influenza pandemic. The repeated searches showed growth in the body of work on testing, contact tracing, vaccines and pharmaceuticals as a resource relevant to COVID-19. This was expected as the understanding of COVID-19 vaccines and pharmaceutical treatment grew over the course of the pandemic, with vaccines being rolled out beginning 2021. Testing schemes also gained traction over the course of the year 2021, with many countries requiring proof of vaccination or negative COVID-19 status on entry or for public venues.

While this is a comprehensive overview, it is by no means exhaustive. Bayram et al. [164] published a Delphi study on critical care resources in 2013, listing 58 different resources possibly required for critical care of pandemic influenza. Data on the availability or consumption of such specific resources as needles, blood pressure cuffs or intravenous pumps will generally not be published in scientific studies but may be known to hospital management. Non-PPE consumables are critical for delivery of care and shortages have occurred during the COVID-19 pandemic [165] so ensuring sufficient stock should be a priority for pandemic planners. However, such resources may be too specific and omitted from resource models to avoid excessive complexity.

This study has multiple strengths. The diverse backgrounds of researchers involved in this study ensured that perspectives from public health, healthcare (primary care and internal medicine), epidemiology, infection prevention and control, and mathematical modelling were all represented in the design and execution of this study. This study was conducted as part of the PANDEM-2 project, which included resource modelling work, ensuring practical application of the findings such as in a functional exercise [12] .

The search strategy is, however, also one of the limitations of this study. As we chose to search for resources in the context of a pandemic, certain resources or resource parameters may not have been identified in this study because publications on those resources were not pandemic-related. The search did not include terms specific for prophylactic or therapeutic drugs. This is a limitation and may have caused under-inclusion of studies on prophylactic or therapeutic drugs if they did not contain other keywords present in our search strategy. Reviews on therapy for pandemic influenza and COVID-19 are widely available [166–171]. Only three studies on contact tracing were included in this review. While we identified more studies on contact tracing, the main focus of these studies was investigating contact tracing as a non-pharmaceutical intervention (NPI) and its effect on reducing morbidity and mortality. We also did not include grey literature or policy documents in this study, which could contain information on resources that is not published scientifically. Users of the data collected in this study should also be aware of the highly diverse context in which these studies are performed, with regional context, population characteristics and timing of the study in relation to pandemic phases being important factors. The final repeat of our search identified a number of reviews on COVID-19 vaccines and diagnostic tests [172–176]. We elected not to include the articles reviewed therein in our work as our aim was to provide an overview of available resource data and not an in-depth review on the efficacy of COVID-19 vaccines or tests.

## Conclusion

In this review, we identified a broad range of public health and healthcare resources and their corresponding parameters that have been published in relation to the 2009 influenza A(H1N1) and COVID-19 pandemics. Each pandemic comes with its own unexpected and unforeseen challenges, caused by specific features related to the pathogen and the disease, but also can be related to the global or geopolitical context at that time, which may impact the availability and accessibility of resources needed for pandemic response. Nevertheless, the data gathered in this study will provide valuable input for resource modelling work and may also be of use to policy makers and pandemic planners. Hospital resources such as non-ICU beds, ICU beds, mechanical ventilation and healthcare staff, vaccines and PPE are well described in scientific literature, while for other resources such as contact tracing, general practitioners and home care, limited data on capacity and use is currently available in scientific literature. More research on mapping the availability and use of these resources would be of great value for preparing for future pandemics by allowing the creation of more realistic scenarios and estimates for resource needs in order to prevent resource shortages and disruption to critical care delivery. Future research could also use the data gathered in this review to estimate required stockpiling of healthcare resources for future pandemics, and to determine the size of pandemic emergency funds needed to cover resource requirements. This will especially apply to potential future pandemics, when countries might find themselves facing shortages of resources. Therefore detailed pandemic planning would contribute health system resilience when the next pandemic confronts us in the future.

## Electronic supplementary material

Below is the link to the electronic supplementary material.

Supplementary Material 1

## Data Availability

All data included in this study are reported in the article or uploaded as supplementary information.
