# Supplementary material for: A systematic literature review on public health and healthcare resources for pandemic preparedness planning
Source: BMC Public Health. 2024 Nov 11;24:3114. doi: 10.1186/s12889-024-20629-z (PMC11552315; doi:10.1186/s12889-024-20629-z)
Supplement: Supplementary file 1 — Supplementary Material 1 [file 12889_2024_20629_MOESM1_ESM.docx]

A systematic literature review on public health and healthcare resources for pandemic preparedness planning

Supplementary material

1. **PRISMA checklist**

| **Section/topic** | **#** | **Checklist item** | **Included?** |
| --- | --- | --- | --- |
| **TITLE** |  | | |
| Title | 1 | Identify the report as a systematic review, meta-analysis, or both. | **√** |
| **ABSTRACT** |  | | |
| Structured summary | 2 | Provide a structured summary including, as applicable: background; objectives; data sources; study eligibility criteria, participants, and interventions; study appraisal and synthesis methods; results; limitations; conclusions and implications of key findings; systematic review registration number. | **√** |
| **INTRODUCTION** |  | | |
| Rationale | 3 | Describe the rationale for the review in the context of what is already known. | **√** |
| Objectives | 4 | Provide an explicit statement of questions being addressed with reference to participants, interventions, comparisons, outcomes, and study design (PICOS). | **√** |
| **METHODS** |  | | |
| Protocol and registration | 5 | Indicate if a review protocol exists, if and where it can be accessed (e.g., Web address), and, if available, provide registration information including registration number. | **n/a** |
| Eligibility criteria | 6 | Specify study characteristics (e.g., PICOS, length of follow-up) and report characteristics (e.g., years considered, language, publication status) used as criteria for eligibility, giving rationale. | **√** |
| Information sources | 7 | Describe all information sources (e.g., databases with dates of coverage, contact with study authors to identify additional studies) in the search and date last searched. | **√** |
| Search | 8 | Present full electronic search strategy for at least one database, including any limits used, such that it could be repeated. | **Suppl. 1** |
| Study selection | 9 | State the process for selecting studies (i.e., screening, eligibility, included in systematic review, and, if applicable, included in the meta-analysis). | **√** |
| Data collection process | 10 | Describe method of data extraction from reports (e.g., piloted forms, independently, in duplicate) and any processes for obtaining and confirming data from investigators. | **√** |
| Data items | 11 | List and define all variables for which data were sought (e.g., PICOS, funding sources) and any assumptions and simplifications made. | **√** |
| Risk of bias in individual studies | 12 | Describe methods used for assessing risk of bias of individual studies (including specification of whether this was done at the study or outcome level), and how this information is to be used in any data synthesis. | **n/a** |
| Summary measures | 13 | State the principal summary measures (e.g., risk ratio, difference in means). | **√** |
| Synthesis of results | 14 | Describe the methods of handling data and combining results of studies, if done, including measures of consistency (e.g., I^2^) for each meta-analysis. | **√** |
| Risk of bias across studies | 15 | Specify any assessment of risk of bias that may affect the cumulative evidence (e.g., publication bias, selective reporting within studies). | **n/a** |
| Additional analyses | 16 | Describe methods of additional analyses (e.g., sensitivity or subgroup analyses, meta-regression), if done, indicating which were pre-specified. | **n/a** |
| **RESULTS** | | |  |
| Study selection | 17 | Give numbers of studies screened, assessed for eligibility, and included in the review, with reasons for exclusions at each stage, ideally with a flow diagram. | **Fig 1** |
| Study characteristics | 18 | For each study, present characteristics for which data were extracted (e.g., study size, PICOS, follow-up period) and provide the citations. | **Table 1, Supp table 1** |
| Risk of bias within studies | 19 | Present data on risk of bias of each study and, if available, any outcome level assessment (see item 12). | **n/a** |
| Results of individual studies | 20 | For all outcomes considered (benefits or harms), present, for each study: (a) simple summary data for each intervention group (b) effect estimates and confidence intervals, ideally with a forest plot. | **Table 2, Supp table 2** |
| Synthesis of results | 21 | Present results of each meta-analysis done, including confidence intervals and measures of consistency. | **n/a** |
| Risk of bias across studies | 22 | Present results of any assessment of risk of bias across studies (see Item 15). | **n/a** |
| Additional analysis | 23 | Give results of additional analyses, if done (e.g., sensitivity or subgroup analyses, meta-regression [see Item 16]). | **n/a** |
| **DISCUSSION** | | |  |
| Summary of evidence | 24 | Summarize the main findings including the strength of evidence for each main outcome; consider their relevance to key groups (e.g., healthcare providers, users, and policy makers). | **√** |
| Limitations | 25 | Discuss limitations at study and outcome level (e.g., risk of bias), and at review-level (e.g., incomplete retrieval of identified research, reporting bias). | **√** |
| Conclusions | 26 | Provide a general interpretation of the results in the context of other evidence, and implications for future research. | **√** |
| **FUNDING** | | |  |
| Funding | 27 | Describe sources of funding for the systematic review and other support (e.g., supply of data); role of funders for the systematic review. | **√** |

1. **Search terms and results**

**First search – 21^st^ of May 2021**

#28 #27 AND ('Article'/it OR 'Article in Press'/it OR 'Review'/it) 1,166

#27 #26 AND [1995-2021]/py 1,492

#26 #25 AND ([dutch]/lim OR [english]/lim) 1,493

#25 #23 OR #24 1,537

#24 #12 AND #22 894

#23 #12 AND #15 658

#22 #16 OR #17 OR #18 OR #19 OR #20 OR #21 129,839

#21 'corona':ti AND ('patient*':ti OR 'disease*':ti OR 'ill*':ti OR 'virus*':ti) 657

#20 'coronavac*':ti OR 'corona vac*':ti OR 'corona-vac*':ti OR 201

(('corona*' NEAR/2 'vaccin*'):ti)

#19 'sars-cov-2*':ti OR 'sars-ncov*':ti OR '2019-ncov*':ti OR 'hcov-19*':ti 17,790

#18 'covid-19*':ti 91,157

#17 '2019 novel coronavirus'/exp/mj 9,513

#16 'coronavirus*':ti OR 'coronavirus disease 2019'/exp/mj 107,699

#15 #13 OR #14 106,316

#14 ('influenz*' NEAR/2 'pandem*'):ti,ab 11,200

#13 'Novel influenza A(H1N1)pdm09'/exp OR 'influenza'/exp/mj OR 'mexican flu':ti,ab OR 105,344

'mexican influenza*':ti,ab OR 'influenza*':ti

#12 #6 AND #11 2,002

#11 #7 OR #8 OR #9 OR #10 2,338,613

#10 'health care utilization'/exp/mj OR 'health care utilizat*':ti OR 507,491

('health care personnel'/exp AND 'workload'/exp) OR 'vaccination'/exp OR

'intensive care'/exp/mj

#9 'health care planning'/exp/mj OR 'health care planning*':ti OR 125,795

'mass screening'/exp/mj

#8 'dashboard*':ti OR 'model*':ti OR 'protocol*':ti OR 'scenario*':ti OR 1,011,242

'simulat*':ti OR 'deplet*':ti OR 'occupanc*':ti OR 'preparedness*':ti

#7 'parameter*':ti OR 'simulation'/exp OR 'decision making'/exp 866,948

#6 #1 AND #5 6,927

#5 #2 OR #3 OR #4 235,472

#4 'resource allocation'/exp OR 'resource allocat*':ti,ab OR 'resourc*':ti OR 96,524

'allocat*':ti OR (('exit*':ti OR 'reopening*':ti OR 'mitigat*':ti) AND 'strateg*':ti) OR

('absenteeism'/exp AND 'workload'/exp) OR 'contact examination'/exp

#3 'resource management'/exp/mj OR 'resource management*':ti,ab OR 53,404

'resource shortage'/exp OR 'resource shortage*':ti,ab OR ('diagnostic

test'/exp/mj AND 'mass screening'/exp) OR 'control measure*':ti,ab OR

'testing capacity'/exp

#2 'resourc*':ti OR 'capacity':ti OR 'hospital bed*':ti OR 'health demand*':ti OR 151,093

'intensive care unit load*':ti,ab OR 'ic load*':ti,ab OR (('personal protective

equipment*' NEAR/2 'estima*'):ti,ab) OR (('ppe*' NEAR/2 'estima*'):ti,ab) OR

'stockpil*':ti,ab OR (('health*' NEAR/2 'burden*'):ti,ab) OR 'preparedness* and

respons*':ti,ab OR 'preparedness* and control':ti,ab OR 'pandemic preparedness*':ti

#1 'pandemic'/exp OR 'pandem*':ti,ab OR 'disease transmission'/exp/mj 139,914

**Updated search – 25^th^ of March 2022**

#28 #27 AND ('Article'/it OR 'Article in Press'/it OR 'Review'/it) 788

#27 #26 AND [2021-2022]/py 1,057

#26 #25 AND ([dutch]/lim OR [english]/lim) 2,241

#25 #23 OR #24 2,296

#24 #12 AND #22 1,639

#23 #12 AND #15 677

#22 #16 OR #17 OR #18 OR #19 OR #20 OR #21 229,489

#21 'corona':ti AND ('patient*':ti OR 'disease*':ti OR 'ill*':ti OR 'virus*':ti) 857

#20 'coronavac*':ti OR 'corona vac*':ti OR 'corona-vac*':ti O 517

R (('corona*' NEAR/2 'vaccin*'):ti)

#19 'sars-cov-2*':ti OR 'sars-ncov*':ti OR '2019-ncov*':ti OR 'hcov-19*':ti 36,428

#18 'covid-19*':ti 163,230

#17 '2019 novel coronavirus'/exp/mj 26,098

#16 'coronavirus*':ti OR 'coronavirus disease 2019'/exp/mj 179,039

#15 #13 OR #14 109,895

#14 ('influenz*' NEAR/2 'pandem*'):ti,ab 11,472

#13 'Novel influenza A(H1N1)pdm09'/exp OR 'influenza'/exp/mj OR 'mexican flu':ti,ab OR 108,839

'mexican influenza*':ti,ab OR 'influenza*':ti

#12 #6 AND #11 2,936

#11 #7 OR #8 OR #9 OR #10 2,486,608

#10 'health care utilization'/exp/mj OR 'health care utilizat*':ti OR 541,429

('health care personnel'/exp AND 'workload'/exp) OR 'vaccination'/exp OR

'intensive care'/exp/mj

#9 'health care planning'/exp/mj OR 'health care planning*':ti OR 130,902

'mass screening'/exp/mj

#8 'dashboard*':ti OR 'model*':ti OR 'protocol*':ti OR 'scenario*':ti OR 1,077,532

'simulat*':ti OR 'deplet*':ti OR 'occupanc*':ti OR 'preparedness*':ti

#7 'parameter*':ti OR 'simulation'/exp OR 'decision making'/exp 922,073

#6 #1 AND #5 10,442

#5 #2 OR #3 OR #4 253,409

#4 'resource allocation'/exp OR 'resource allocat*':ti,ab OR 'resourc*':ti OR 103,958

'allocat*':ti OR (('exit*':ti OR 'reopening*':ti OR 'mitigat*':ti) AND 'strateg*':ti) OR

('absenteeism'/exp AND 'workload'/exp) OR 'contact examination'/exp

#3 'resource management'/exp/mj OR 'resource management*':ti,ab OR 58,417

'resource shortage'/exp OR 'resource shortage*':ti,ab OR ('diagnostic

test'/exp/mj AND 'mass screening'/exp) OR 'control measure*':ti,ab OR 'testing capacity'/exp

#2 'resourc*':ti OR 'capacity':ti OR 'hospital bed*':ti OR 'health demand*':ti OR 161,258

'intensive care unit load*':ti,ab OR 'ic load*':ti,ab OR (('personal protective

equipment*' NEAR/2 'estima*'):ti,ab) OR (('ppe*' NEAR/2 'estima*'):ti,ab) OR 'stockpil*':ti,ab OR (('health*' NEAR/2 'burden*'):ti,ab) OR 'preparedness* and respons*':ti,ab OR 'preparedness* and control':ti,ab OR 'pandemic preparedness*':ti

#1 'pandemic'/exp OR 'pandem*':ti,ab OR 'disease transmission'/exp/mj 206,659

**Updated search – 3^rd^ of June 2023**

#28 #27 AND ('article'/it OR 'article in press'/it OR 'review'/it) 758
#27 #26 AND [2022-2023]/py 991
#26 #25 AND ([dutch]/lim OR [english]/lim) 3116
#25 #23 OR #24 3182
#24 #12 AND #22 2485
#23 #12 AND #15 750

#22 #16 OR #17 OR #18 OR #19 OR #20 OR #21 352854
#21 'corona':ti AND ('patient*':ti OR 'disease*':ti OR 'ill*':ti OR 'virus*':ti) 1115
#20 'coronavac*':ti OR 'corona vac*':ti OR 'corona-vac*':ti OR (('corona*' NEAR/2 'vaccin*'):ti) 1020
#19 'sars-cov-2*':ti OR 'sars-ncov*':ti OR '2019-ncov*':ti OR 'hcov-19*':ti 60260

#18 'covid-19*':ti 251014

#17 '2019 novel coronavirus'/exp/mj 41877

#16 'coronavirus*':ti OR 'coronavirus disease 2019'/exp/mj 275331

#15 #13 OR #14 118017

#14 ('influenz*' NEAR/2 'pandem*'):ti,ab 11810

#13 'pandemic influenza'/exp OR 'influenza'/exp/mj OR 'mexican flu':ti,ab OR 'mexican influenza*':ti,ab OR 'influenza*':ti 117084

#12 #6 AND #11 4166

#11 #7 OR #8 OR #9 OR #10 2741445

#10 'health care utilization'/exp/mj OR 'health care utilizat*':ti OR ('health care personnel'/exp AND 'workload'/exp) OR 'vaccination'/exp OR 'intensive care'/exp/mj 599370

#9 'health care planning'/exp/mj OR 'health care planning*':ti OR 'mass screening'/exp/mj 139472

#8 'dashboard*':ti OR 'model*':ti OR 'protocol*':ti OR 'scenario*':ti OR 'simulat*':ti OR 'deplet*':ti OR 'occupanc*':ti OR 'preparedness*':ti 1187025

#7 'parameter*':ti OR 'simulation'/exp OR 'decision making'/exp 1026060

#6 #3 AND #5 14509

#5 #1 OR #2 OR #4 280992

#4 'resource allocation'/exp OR 'resource allocat*':ti,ab OR 'resourc*':ti OR 'allocat*':ti OR (('exit*':ti OR 'reopening*':ti OR 'mitigat*':ti) AND 'strateg*':ti) OR ('absenteeism'/exp AND 'workload'/exp) OR 'contact examination'/exp 115257

#3 'pandemic'/exp OR 'pandem*':ti,ab OR 'disease transmission'/exp/mj 294079

#2 'resource management'/exp/mj OR 'resource management*':ti,ab OR 'resource shortage'/exp OR 'resource shortage*':ti,ab OR ('diagnostic test'/exp/mj AND 'mass screening'/exp) OR 'control measure*':ti,ab OR 'testing capacity'/exp 65910

#1 'resourc*':ti OR 'capacity':ti OR 'hospital bed*':ti OR 'health demand*':ti OR 'intensive care unit load*':ti,ab OR 'ic load*':ti,ab OR (('personal protective equipment*' NEAR/2 'estima*'):ti,ab) OR (('ppe*' NEAR/2 'estima*'):ti,ab) OR 'stockpil*':ti,ab OR (('health*' NEAR/2 'burden*'):ti,ab) OR 'preparedness* and respons*':ti,ab OR 'preparedness* and control':ti,ab OR 'pandemic preparedness*':ti 177669

**Supplementary table 1.** **Overview of all studies included in this systematic review**

| **Study** | **Year** | **Country** | **World Bank classification** | **Study period** | **Study aproach** | **Disease type** | **Research focus** |
| --- | --- | --- | --- | --- | --- | --- | --- |
| Aaby[1] | 2006 | United States | High income | 2005 | Model building | Pandemic influenza | Emergency preparedness |
| Abd El Ghaffar[2] | 2021 | Egypt | Lower middle income | 2020 | Observational | COVID-19 | Hospital |
| Abdullah[3] | 2021 | Singapore | High income | 2019-2020 | Model building | COVID-19 | Hospital |
| Abedrabboh[4] | 2021 | United Kingdom | High income | 2020 | Model building | COVID-19 | Hospital |
| Abramovich[5] | 2017 | United States | High income | 2015 | Model building | Pandemic influenza | Hospital |
| Adisasmito[6] | 2015 | Indonesia | Upper middle income | 2015 | Model building | Pandemic influenza | Hospital |
| Alexander[7] | 2021 | United States | High income | 2020 | Evaluation | COVID-19 | Public health |
| Althobaity[8] | 2022 | Multiple^A^ | High income | 2020 | Model building | COVID-19 | Vaccination |
| Ang[9] | 2009 | Singapore | High income | 2009 | Evaluation | Pandemic influenza | Hospital |
| Arya[10] | 2020 | Canada | High income | 2019 | Evaluation | COVID-19 | Hospital |
| Avelino-Silva[11] | 2023 | Brazil | Upper middle income | 2020 | Observational | COVID-19 | Hospital |
| Bae[12] | 2021 | Israel | High income | 2020 | Observational | COVID-19 | Hospital |
| Bagshaw[13] | 2023 | Canada | High income | 2021 | Observational | COVID-19 | Hospital |
| Bagshaw[14] | 2022 | Canada | High income | 2021-2022 | Observational | COVID-19 | Hospital |
| Baik[15] | 2022 | India | Lower middle income | 2020 | Model building | COVID-19 | Testing strategies |
| Baker[16] | 2011 | Australia | High income | 2009 | Model building | Pandemic influenza | Hospital |
| Baldi[17] | 2020 | Italy | High income | 2019-2020 | Evaluation | COVID-19 | First responder |
| Balicer[18] | 2005 | Israel | High income | 1999 | Cost-benefit analysis | Pandemic influenza | Hospital |
| Barrett[19] | 2020 | Canada | High income | 2020 | Model building | COVID-19 | Hospital |
| Bartoszko[20] | 2021 | Canada | High income | 2020 | Observational | COVID-19 | Hospital |
| Beatty[21] | 2021 | Ireland | High income | 2020 | Observational | COVID-19 | Hospital |
| Beijaert[22] | 2021 | The Netherlands | High income | 2021 | Survey | COVID-19 | General practice |
| Berger[23] | 2022 | Multiple^B^ | High income | 2020 | Observational | COVID-19 | Hospital |
| Booton[24] | 2021 | United Kingdom | High income | 2020 | Model building | COVID-19 | Hospital |
| Boussarsar[25] | 2023 | Tunisia | Lower middle income | 2020-2022 | Observational | COVID-19 | Hospital |
| Braeye[26] | 2023 | Belgium | High income | 2021-2022 | Model building | COVID-19 | Vaccination |
| Canga[27] | 2022 | Spain | High income | 2020-2021 | Model building | COVID-19 | Vaccination |
| Castagna[28] | 2022 | United States | High income | 2020-2021 | Observational | COVID-19 | Hospital |
| Cattaneo[29] | 2022 | Italy | High income | 2020-2021 | Model building | COVID-19 | Vaccination |
| Chalk[30] | 2021 | United Kingdom | High income | 2020 | Model building | COVID-19 | Hospital |
| Challen[31] | 2007 | United States & England | High income | 2005 | Evaluation | Pandemic influenza | Hospital |
| Chappell[32] | 2020 | United Kingdom | High income | 2020 | Clinical trial | COVID-19 | Hospital |
| Chen[33] | 2021 | United States | High income | 2020 | Model building | COVID-19 | Hospital |
| Chhim[34] | 2023 | Cambodia | Lower middle income | 2020-2022 | Evaluation | COVID-19 | Emergency preparedness |
| Chomton[35] | 2021 | France | High income | 2020 | Observational | COVID-19 | Hospital |
| Chu[36] | 2011 | South Korea | High income | 2011 | Model building | Pandemic influenza | Hospital |
| Chua[37] | 2021 | Singapore | High income | 2021 | Evaluation | COVID-19 | Public health |
| Considine[38] | 2011 | Australia | High income | 2009 | Survey | Pandemic influenza | Hospital |
| Cruz-Aponte[39] | 2011 | United States | High income | 2006-2011 | Model building | Pandemic influenza | Vaccination |
| Cummings[40] | 2020 | United States | High income | 2020 | Observational | COVID-19 | Hospital |
| Dadhwal[41] | 2022 | United Kingdom | High income | 2019-2020 | Observational | COVID-19 | Hospital |
| Datta[42] | 2022 | United States | High income | 2020-2021 | Model building | COVID-19 | Hospital |
| Davis[43] | 2021 | United Kingdom | High income | 2020-2021 | Model building | COVID-19 | Contact tracing |
| de Léon[44] | 2021 | United States | High income | 2021 | Model building | COVID-19 | Vaccination |
| De Wolff[45] | 2021 | Multiple^C^ | High income | 2020 | Model building | COVID-19 | Testing strategies |
| Doron[46] | 2023 | United States | High income | 2022 | Observational | COVID-19 | Hospital |
| Doyle[47] | 2006 | France | High income | 2006 | Model building | Pandemic influenza | Hospital |
| Elhadi[48] | 2021 | Libya | Upper middle income | 2020 | Observational | COVID-19 | Hospital |
| Elhakim[49] | 2022 | Djibouti | Lower middle income | 2020-2021 | Evaluation | COVID-19 | Emergency preparedness |
| Ercole[50] | 2009 | United Kingdom | High income | 2009 | Model building | Pandemic influenza | Hospital |
| Eyre[51] | 2023 | United Kingdom | High income | 2020-2022 | Observational | COVID-19 | Testing strategies |
| Fort[52] | 2020 | United States | High income | 2020 | Model building | COVID-19 | Hospital |
| Galante[53] | 2012 | Spain | High income | 2009-2010 | Observational | Pandemic influenza | Hospital |
| Gelfman[54] | 2022 | United States | High income | 2020 | Observational | COVID-19 | Hospital |
| Giannakeas[55] | 2020 | United States & Canada | High income | 2020 | Model building | COVID-19 | Hospital |
| Godeaux[56] | 2015 | United States | High income | 2013 | Clinical trial | Pandemic influenza | Vaccination |
| Grasselli[57] | 2020 | Italy | High income | 2020 | Observational | COVID-19 | Hospital |
| Haase[58] | 2022 | Denmark | High income | 2020 | Observational | COVID-19 | Hospital |
| Hara[59] | 2002 | Japan | High income | 1999 | Evaluation | Pandemic influenza | Hospital |
| Hashikura[60] | 2009 | Japan | High income | 1950-2008 | Model building | Pandemic influenza | Hospital |
| Hayden[61] | 2021 | Italy | High income | 2020 | Observational | COVID-19 | Hospital |
| Hertzberg[62] | 2021 | Sweden | High income | 2020 | Observational | COVID-19 | Hospital |
| Ho[63] | 2022 | Taiwan | High income | 2009-2010 & 2011-2012 | Observational | Pandemic influenza | School |
| Hohl[64] | 2022 | Canada | High income | 2020 | Observational | COVID-19 | Hospital |
| Hong[65] | 2022 | South Korea | High income | 2021-2022 | Evaluation | COVID-19 | Vaccination |
| Hsu[66] | 2021 | Germany | High income | 2020-2021 | Observational | COVID-19 | Public health |
| Huang[67] | 2017 | United States | High income | 2005-2009 | Model building | Pandemic influenza | Hospital |
| Iragorri[68] | 2020 | Colombia | Upper middle income | 2016-2020 | Model building | COVID-19 | Hospital |
| Irvine[69] | 2021 | United Kingdom | High income | 2020 | Model building | COVID-19 | Hospital |
| Islam[70] | 2021 | Bangladesh | Lower middle income | 2020 | Observational | COVID-19 | Hospital |
| Jost[71] | 2020 | France | High income | 2020 | Evaluation | COVID-19 | First responder |
| Kafan[72] | 2021 | Iran | Upper middle income | 2020 | Observational | COVID-19 | Hospital |
| Kasaie[73] | 2013 | United States | High income | 2009 | Model building | Pandemic influenza | Hospital |
| Kasturi[74] | 2021 | United States | High income | 2018-2020 | Model building | COVID-19 | Hospital |
| Khawaja[75] | 2021 | Pakistan | Lower middle income | 2019-2020 | Observational | COVID-19 | Hospital |
| Kriegova[76] | 2021 | Czech Republic | High income | 2020 | Observational | COVID-19 | Hospital |
| Krumkamp[77] | 2011 | Thailand | Upper middle income | 2009 | Model building | Pandemic influenza | Hospital |
| Laake[78] | 2021 | Norway | High income | 2020 | Observational | COVID-19 | Hospital |
| Lam[79] | 2022 | Singapore | High income | 2020 | Model building | COVID-19 | Hospital |
| Lawandi[80] | 2022 | United States | High income | 2020-2021 | Observational | COVID-19 | Hospital |
| Lee[81] | 2013 | South Korea | High income | 2013 | Evaluation | Pandemic influenza | Emergency preparedness |
| Lee[82] | 2007 | Singapore | High income | 2000-2005 | Model building | Pandemic influenza | Hospital |
| Lee[83] | 2009 | United States | High income | 2000-2009 | Model building | Pandemic influenza | Hospital |
| Lee[84] | 2010 | United States | High income | 2004-2006 | Model building | Pandemic influenza | Hospital |
| Li[85] | 2022 | Global | High/upper middle/lower middle/lower income | 2020-2021 | Model building | COVID-19 | Vaccination |
| Mahévas[86] | 2020 | France | High income | 2020 | Clinical trial | COVID-19 | Hospital |
| Marijon[87] | 2020 | France | High income | 2020 | Observational | COVID-19 | First responder |
| Mayorga[88] | 2020 | Argentina | Upper middle income | 2020 | Model building | COVID-19 | Hospital |
| McCabe[89] | 2021 | France, Germany & Italy | High income | 2020 | Model building | COVID-19 | Hospital |
| Medema[90] | 2004 | United Kingdom | High income | 1918-1919 & 1994-2002 | Model building | Pandemic influenza | Emergency preparedness |
| Melman[91] | 2021 | United Kingdom | High income | 2020 | Model building | COVID-19 | Hospital |
| Merler[92] | 2009 | Italy | High income | 2009 | Model building | Pandemic influenza | Public health |
| Methi[93] | 2022 | Norway | High income | 2020-2022 | Observational | COVID-19 | Hospital |
| Miller[94] | 2008 | United States | High income | 2008 | Model building | Pandemic influenza | Hospital |
| Milne[95] | 2010 | Australia | High income | 2010 | Model building | Pandemic influenza | Vaccination |
| Modisenyane[96] | 2022 | South-Africa | Upper middle income | 2020-2021 | Model building | COVID-19 | Contact tracing & testing strategies |
| Moon[97] | 2022 | United States | High income | 2020-2021 | Observational | COVID-19 | Hospital |
| Nap[98] | 2008 | The Netherlands | High income | 2007 | Model building | Pandemic influenza | Hospital |
| Nap[99] | 2007 | The Netherlands | High income | 2007 | Model building | Pandemic influenza | Hospital |
| Nicolay[100] | 2010 | Ireland | High income | 2009 | Evaluation | Pandemic influenza | Hospital |
| Noreen[101] | 2020 | Pakistan | Lower middle income | 2020 | Observational | COVID-19 | Public health |
| Nuño[102] | 2007 | United States, United Kingdom & The Netherlands | High income | 2007 | Model building | Pandemic influenza | Public health |
| Oakley[103] | 2020 | United Kingdom | High income | 2020 | Evaluation | COVID-19 | Hospital |
| Opstelten[104] | 2007 | The Netherlands | High income | 2001-2006 | Evaluation | Pandemic influenza | Emergency preparedness |
| Pastorino[105] | 2021 | Italy | High income | 2020 | Observational | COVID-19 | Hospital |
| Pham[106] | 2021 | The Netherlands | High income | 2020 | Model building | COVID-19 | Hospital |
| Philips[107] | 2022 | United States | High income | 2020 | Observational | COVID-19 | Hospital |
| Phin[108] | 2009 | United Kingdom | High income | 2009 | Model building | Pandemic influenza | Hospital |
| Prada[109] | 2022 | Colombia | Upper middle income | 2020-2022 | Evaluation | COVID-19 | Emergency preparedness |
| Rainisch[110] | 2022 | United States | High income | 2020-2021 | Model building | COVID-19 | Contact tracing |
| Ravikumar[111] | 2020 | India | Lower middle income | 2020 | Evaluation | COVID-19 | Hospital |
| Rennert-May[112] | 2023 | United States | High income | 2020-2021 | Observational | COVID-19 | Hospital |
| Richardson[113] | 2020 | United States | High income | 2020 | Observational | COVID-19 | Hospital |
| Ritter[114] | 2021 | Germany, Italy & Spain | High income | 2020 | Model building | COVID-19 | Hospital |
| Rosenberg[115] | 2020 | United States | High income | 2020 | Clinical trial | COVID-19 | Hospital |
| Ruggeri[116] | 2022 | Portugal | High income | 2021 | Model building | COVID-19 | Hospital |
| Ruggeri[117] | 2022 | Saudi Arabia | High income | 2021 | Model building | COVID-19 | Hospital |
| Sander[118] | 2009 | United States | High income | 2007 | Model building | Pandemic influenza | Public health |
| Saunders-Hastings[119] | 2017 | Canada | High income | 2009-2014 | Model building | Pandemic influenza | Hospital |
| Scott[120] | 2022 | United States | High income | 2020 | Observational | COVID-19 | Hospital |
| Sharma[121] | 2022 | United States | High income | 2020-2021 | Observational | COVID-19 | Public health |
| Smetanin[122] | 2009 | Canada | High income | 2009 | Model building | Pandemic influenza | Hospital |
| Solanki[123] | 2022 | South-Africa | Upper middle income | 2020-2021 | Observational | COVID-19 | Hospital |
| Sritipsukho[124] | 2022 | Thailand | Upper middle income | 2021 | Observational | COVID-19 | Vaccination |
| Stanislawski[125] | 2023 | Germany | Upper middle income | 2020 | Observational | COVID-19 | Testing strategies |
| Stein[126] | 2012 | Multiple^D^ | Upper/lower middle income | 2009 | Model building | Pandemic influenza | Hospital |
| Stepanova[127] | 2022 | United States | High income | 2020-2022 | Observational | COVID-19 | Hospital |
| Stiff[128] | 2011 | Canada | High income | 2009 | Model building | Pandemic influenza | Hospital |
| Subiros[129] | 2022 | France | High income | 2020-2021 | Observational | COVID-19 | Hospital |
| Suetens[130] | 2021 | Multiple^E^ | High income | 2021 | Observational | COVID-19 | Long-term care facilities |
| Swaminathan[131] | 2007 | Australia | High income | 2007 | Model building | Pandemic influenza | Hospital |
| Taboe[132] | 2023 | United States | High income | 2021-2022 | Model building | COVID-19 | Vaccination |
| Ten Eyck[133] | 2008 | United States | High income | 2008 | Model building | Pandemic influenza | Hospital |
| Tran Kiem[134] | 2021 | France | High income | 2020 | Model building | COVID-19 | Hospital |
| Trentini[135] | 2022 | Italy | High income | 2020 | Observational | COVID-19 | Hospital |
| Tuite[136] | 2010 | Canada | High income | 2009 | Model building | Pandemic influenza | Hospital |
| Van Genugten[137] | 2004 | The Netherlands | High income | 2002 | Model building | Pandemic influenza | Hospital |
| Verma[138] | 2020 | India | Lower middle income | 2020 | Model building | COVID-19 | Hospital |
| Vidal-Cortés[139] | 2022 | Spain | High income | 2021 | Observational | COVID-19 | Hospital |
| Wang[140] | 2020 | China | Upper middle income | 2020 | Clinical trial | COVID-19 | Hospital |
| Webb[141] | 2022 | Estonia, Latvia, and Lithuania | High income | 2020 | Evaluation | COVID-19 | Emergency preparedness |
| Weissman[142] | 2020 | United States | High income | 2020 | Model building | COVID-19 | Hospital |
| Wood[143] | 2009 | Australia | High income | 2007 | Model building | Pandemic influenza | Vaccination |
| Wood[144] | 2020 | United Kingdom | High income | 2020 | Model building | COVID-19 | Hospital |
| Zhang[145] | 2021 | China | Upper middle income | 2020 | Evaluation | COVID-19 | Hospital |
| Zhang[146] | 2006 | United States | High income | 2006 | Model building | Pandemic influenza | Hospital |
| Zirbes[147] | 2023 | Germany | High income | 2020-2021 | Observational | COVID-19 | Testing strategies |

***Supplementary table 2. Data extracted on PPE use, pharmaceuticals, testing and tracing and other resources.***

|  | **Pandemic influenza** | **COVID-19** |
| --- | --- | --- |
| **PPE** |  | |
| Number of studies | 6 | 2 |
| Usage | 91 surgical masks per hospitalized patient 40 N95 masks per hospitalized patient [6] | Use of one kit per patient: demand of 16.2-18.5 kits per occupied bed, per day Use of one kit per session: demand of 10.7-12.9 kits per occupied bed, per day [4] |
|  | For high risk procedures (intubation, suctioning before intubation, manipulating the oxygen mask, and others) (PPE needed per day): - 12 N95 masks (with exhalation valve) - 12 Surgical masks - 12 Goggles - 12 Gowns - 38 Gloves[60] |  |
|  | Hospital of 29 bed (100% occupied) needs per 24h: Surgical masks: 650 Gloves: 600 Disposable aprons: 750 Gowns: 13 FFP3 respirators: 13 Eye goggles: 13 Visor: 1 [108] |  |
|  | PPE needed per day per case: Masks N-95 / N-99: 1 Surgical masks: 5 Face shields: 1 Gloves (pairs): 4 Coverall gowns: 3 [126] |  |
|  | 20-25 PPE sets per patient in first 6 hours.  Mean 25.1 pairs of gloves per patients in the first 6 hours Mean 19.4 N95 masks per patient in the first 6 hours Mean 22.1 gowns per patient in first 6 hours [131] |  |
| Stockpile | Required for hospital of 300 beds:  - 10.528 N95 mask (with  exhalation valve)  - 8.848 N95 masks  - 132.272 surgical masks  - 21.280 goggles  - 21.280 gowns  - 35.056 aprons  - 172.704 pairs of gloves [60] |  |
| Surge demand | - | Surgical masks: 355%, surgical gowns: 1105.6%, plastic aprons: 442.7%, hand sanitizer: 136% [75] |
| **GP** |  | |
| Number of studies | 2 |  |
| Pandemic related GP visits | 12% of population [99] 60.5% of hospitalized patients  One patient visited an average of 2.1 times [53] | - |
| **Ambulances** |  | |
| Number of studies | 0 | 3 |
| Arrival time (increase in minutes) | - | 5-15 [17, 71, 87] |
| **Testing** |  | |
| Number of studies | 0 | 16 |
| Processing time in laboratory (hours) | - | 5 [45] |
| Time to test result (hours) | - | PCR: 24 [15, 121]  Antigen: 1-24 [15, 121] |
| Total amount of tests performed | - | \| UK: 17.6 tests per 100000; US: 44.4; Singapore: 51.4; Italy: 76.2; Germany: 147.9, PCR [45] \| \| --- \| \| US: 2 million tests per day, PCR [7] \| \| Veteran’s Health Administration peak capacity PCR: 351646 per month, 11721 per day; Peak capacity antigen: 90059 per month, 3002 per day [121] \| \| Pakistan: 11 per 100000, PCR [101] \| \| Delhi: 1500 tests per day, PCR [15] \| \| Cambodja: 500 per day (April 2020) to 15000 per day (July 2021), PCR [34] \| \| Djibouti: 419 per day, PCR [49] \| \| South Africa: 10000-15000 per day, PCR [96] \| \| Colombia: 43000 per day (2020), 92070 per day (2022), PCR [109] \| \| Mobile container facility: 137 PCR per day, 28 antigen per day [125] \| \| Mayotte: 1070 per day (PCR), 635 per day (LFA) [129]  Lithuania: 87 tests per 100000 population; Latvia: 178; Estonia: 217 (March 2020); 535-1700 tests per 100000 (May 2020), 2000+ tests per 100000 pop (November 2020) [141] \| \|  \| |
| Test sensitivity | - | PCR: 0.95-0.99 [15, 43, 45, 76]  Antigen: 0.50-0.8 [15, 43, 51, 147]  Antigen during Alpha: 55.7%; Delta: 64.0%; Omicron; 73.0%; for unvaccinated persons: 57.3%; one dose: 67.6%; two+ doses: 69.7%; for symptomatic persons: 68.7%; asymptomatic: 52.8% [51] |
| Test specificity | - | PCR: 0.99[15, 76]  Antigen: [15, 51, 147] |
| **Contact tracing** |  | |
| Number of studies | 0 | 3 |
| Adherence to isolation measures | **-** | 18.2 % adhered to isolation following symptoms and notification [43] |
| Success rate | **-** | Chance of tracing individual: 99.5% within same day for household contacts, 85% within 2 days for school place contacts, 60% within 2 days for workplace contacts, 10% within 3 days for community contacts [29] |
|  | **-** | Cases interviewed: 49%; interviewed cases who named contacts: 25%; contacts who were notified: 59%; contacts who were monitored: 32%; [110] |
| Speed | **-** | Testing to case interview: 3.5 days; testing to contact notification: 4 days [110] |
| **Palliative care** |  | |
| Number of studies | 0 | 2 |
| Palliative care kit contents | - | 1 kit per patient:  - 10 vials Hydromorphone 2 mg/mL  - 10 vials Haloperidol 5 mg/mL  - 10 vials Midazolam 5 mg/mL  - 10 vials Scopolamine 0.4 mg/mL  - 10 vials Acetaminophen 650 mg suppositories  - 1 Foley catheter 16 French  - 10 Mouth swabs  - Subcutaneous cannulae [10] |
| Palliative care need | **-** | 22.3% of hospitalized patients [30] |
| Duration (days) | **-** | 2 [30] |
| **Antivirals, antibiotics and other pharmaceuticals** |  | |
| Number of studies | 10 | 11 |
| Oseltamivir dose | 2 doses per day for 5 days (treatment) [6] | - |
|  | Adults & adolescents aged >_ 13 years: 75 mg bd.  Children aged >_ 12 months, by weight:  15 kg: 30 mg bd. 15-23 kg: 45 mg bd.  24-40 kg: 60 mg bd.  > 40 kg: 75 mg bd.  Children aged 6-11 months: 25 mg bd.  Children aged 3-5 months: 20 mg bd.  Children aged < 3 months: 12 mg bd. [9] |  |
|  | 75 mg/day for prophylaxis and 150 mg/day for treatment [18] |  |
|  | Prophylactic: One dose per day for 7 days. 4 doses 5-6 years, 6 doses 7-12 years, 7 doses for over 12 years.  Treatment: Two doses per day for 5 days. 4 doses 1-3 years, 6 doses for 4-6 years, 8 doses for 7-12 years old, 10 doses for over 12 years. [47] |  |
|  | Standard treatment regimen 75 mg, twice per day for 5 days Prophylaxis required 75 mg once per day for as long as planned [82] |  |
| Zanamivir dose | Adults: 2 x 5 mg inhalations bd for 5 days.  Children aged > 6 years: same as adult dose. [9] | - |
| Amantadine dose | Adults: 200 mg daily in 1-2 doses. Children aged 1-9 years: 5-8 mg/kg daily (maximum 150 mg) in 1-2 doses.  Children aged > 9 years: same as adult dose. [9] | - |
| Efficacy Oseltamivir | 0.61 [83] | - |
|  | Prophylactic: 68-89% protection against infection Treatment: reduction of days with symptoms with 0.5-1.5 days [104] |  |
|  | 60% reduction of incidence of symptomatic disease given infection 30% reduction in incidence infection given exposure [118] |  |
| Efficacy Zanamivir | 0.62 [83] | - |
|  | Prophylactic: 61% protection against infection Treatment: reduction of days with symptoms with 1-2 days [104] |  |
| Efficacy Oseltamivir & Zanamivir | Preexposure prophylaxis 50 days: 71% Postexposure prophylaxis 7 days: 36%  Antiviral therapy: Reduction hospitalizations: 59% Reduction antimicrobial drug use: 63% [18] |  |
| Efficacy Amantadine | 0.61 [83] | - |
| Efficacy Rimantadine | 0.72 [83] | - |
| Stockpile | 4-25% of population [36, 92] | - |
| Remdesivir dose | - | 200 mg day 1, 100 mg days 2-10 [140]  1 dose per day up to 10 days [37] |
| Remdesivir use (% of patients) | - | 4.5% of ICU COVID-19 patients [48]  2.8% of all COVID-19 hospitalizations [80] |
| Remdesivir effect | - | Relative reduction in progression to ICU: 30% [116] |
| General antiviral use | - | 6,7% of all COVID-19 hospitalizations (first wave), 1,5% (second wave) [64] |
| Dexamethasone dose | - | 1 (6 mg) dose per day up to 10 days [32, 37] |
| Dexamethasone use rate | - | 2.2% of COVID-19 patients [54]  34% of COVID-19 hospital admissions [46] |
| Systemic steroid use rate | - | 9.5% of all COVID-19 hospitalizations during the first wave, 28% during the second wave [64]  11.1% of all COVID-19 hospitalizations [80] |
| Amoxicillin dose | 3 capsules per day for 5 days [6] | - |
| Co-trimoxazole dose | 3 capsules per day for 6.33 days [6] | - |
| Hydroxychloroquine dose | - | 200 mg, 400 mg or 600 mg per dose, once or twice per day [115]  600 mg per day [86] |
| Hydroxychloroquine effect | - | No significant differences in in-hospital mortality between patients receiving hydroxychloroquine treatment and control group [115]  In hospitalized COVID-19 pneumonia patients receiving oxygen, no reduction in ICU admissions or deaths 21 days after admission [86]  In hospitalized COVID-19 patients without acute respiratory distress syndrome, no effect on survival 21 days after admission[86] |

**References**

1. Aaby K, Abbey RL, Herrmann JW, Treadwell M, Jordan CS, Wood K. Embracing computer modeling to address pandemic influenza in the 21st century. Journal of Public Health Management and Practice. 2006;12(4):365-72.

2. Abd El Ghaffar MM, Salem MR, Al Soda MF, Abd El Razik MS, Tahoon MH, Tahoon MF, et al. COVID-19 Pandemic Preparedness in Egypt's Teaching Hospitals: A Needs Assessment Study. Frontiers in public health. 2021;9:748666.

3. Abdullah HR, Lam SSW, Ang BY, Pourghaderi A, Nguyen FNHL, Matchar DB, et al. Resuming elective surgery after COVID-19: A simulation modelling framework for guiding the phased opening of operating rooms. International Journal of Medical Informatics. 2022;158.

4. Abedrabboh K, Pilz M, Al-Fagih Z, Al-Fagih OS, Nebel JC, Al-Fagih L. Game theory to enhance stock management of Personal Protective Equipment (PPE) during the COVID-19 outbreak. PLoS ONE. 2021;16(2 February).

5. Abramovich MN, Hershey JC, Callies B, Adalja AA, Tosh PK, Toner ES. Hospital influenza pandemic stockpiling needs: A computer simulation. American Journal of Infection Control. 2017;45(3):272-7.

6. Adisasmito W, Hunter BM, Krumkamp R, Latief K, Rudge JW, Hanvoravongchai P, Coker RJ. Pandemic influenza and health system resource gaps in Bali: an analysis through a resource transmission dynamics model. Asia-Pacific journal of public health / Asia-Pacific Academic Consortium for Public Health. 2015;27(2):NP713-NP33.

7. Alexander M, Unruh L, Koval A, Belanger W. United States response to the COVID-19 pandemic, January-November 2020. Health Economics, Policy and Law. 2022;17(1):62-75.

8. Althobaity Y, Wu J, Tildesley MJ. Non-pharmaceutical interventions and their relevance in the COVID-19 vaccine rollout in Saudi Arabia and Arab Gulf countries. Infectious Disease Modelling. 2022;7(3):545-60.

9. Ang B, Archuleta S, Chiew YF, Chlebicki MP, Chua A, Fisher DA, et al. Management of novel influenza epidemics in Singapore: Consensus recommendations from the Hospital Influenza Workgroup (Singapore). Singapore Medical Journal. 2009;50(6):567-80.

10. Arya A, Buchman S, Gagnon B, Downar J. Pandemic palliative care: Beyond ventilators and saving lives. CMAJ. 2020;192(15):E400-E4.

11. Avelino-Silva VI, Avelino-Silva TJ, Aliberti MJR, Ferreira JC, Cobello Junior V, Silva KR, et al. Prediction of intensive care admission and hospital mortality in COVID-19 patients using demographics and baseline laboratory data. Clinics. 2023;78.

12. Bae J, Kapse S, Singh G, Gattu R, Ali S, Shah N, et al. Predicting mechanical ventilation and mortality in covid-19 using radiomics and deep learning on chest radiographs: A multi-institutional study. Diagnostics. 2021;11(10).

13. Bagshaw SM, Abbott A, Beesoon S, Bowker SL, Zuege DJ, Thanh NX. A population-based assessment of avoidable hospitalizations and resource use of non-vaccinated patients with COVID-19. Canadian journal of public health = Revue canadienne de sante publique. 2023.

14. Bagshaw SM, Abbott A, Beesoon S, Zuege DJ, Wasylak T, Manns B, Nguyen TX. Avoidable intensive care unit resource use and costs of unvaccinated patients with COVID-19: a historical population-based cohort study

Utilisation et coûts évitables des ressources des unités de soins intensifs pour les patients non vaccinés atteints de COVID-19 : une étude de cohorte historique basée sur la population. Canadian Journal of Anesthesia. 2022;69(11):1399-404.

15. Baik Y, Cilloni L, Kendall E, Dowdy D, Arinaminpathy N. Symptom-based vs asymptomatic testing for controlling SARS-CoV-2 transmission in low- and middle-income countries: A modelling analysis. Epidemics. 2022;41.

16. Baker PRA, Sun J, Morris J, Dines A. Epidemiologic modeling with Flusurge for pandemic (H1N1) 2009 outbreak, Queensland, Australia. Emerging Infectious Diseases. 2011;17(9):1608-14.

17. Baldi E, Sechi GM, Mare C, Canevari F, Brancaglione A, Primi R, et al. Out-of-hospital cardiac arrest during the COVID-19 outbreak in Italy. New England Journal of Medicine. 2020;383(5):496-8.

18. Balicer RD, Huerta M, Davidovitch N, Grotto I. Cost-benefit of stockpiling drugs for influenza pandemic. Emerging Infectious Diseases. 2005;11(8):1280-2.

19. Barrett K, Khan YA, Mbiotech SM, Ximenes R, Naimark DMJ, Sander B. Estimation of covid-19induced depletion of hospital resources in ontario, Canada. CMAJ. 2020;192(24):E640-E6.

20. Bartoszko J, Dranitsaris G, Wilcox ME, Del Sorbo L, Mehta S, Peer M, et al. Development of a repeated-measures predictive model and clinical risk score for mortality in ventilated COVID-19 patients

Mise au point d’un modèle prédictif à mesures répétées et d’un score de risque clinique de mortalité pour les patients COVID-19 ventilés. Canadian Journal of Anesthesia. 2022;69(3):343-52.

21. Beatty K, Kavanagh PM. A retrospective cohort study of outcomes in hospitalised COVID-19 patients during the first pandemic wave in Ireland. Irish Journal of Medical Science. 2021.

22. Beijaert RPH, Numans ME. Uitval van huisartsenzorg door covid-19-vaccin Leerpunten voor toekomstige vaccinatierondes. Nederlands Tijdschrift voor Geneeskunde. 2021;165(37).

23. Berger E, Winkelmann J, Eckhardt H, Nimptsch U, Panteli D, Reichebner C, et al. A country-level analysis comparing hospital capacity and utilisation during the first COVID-19 wave across Europe. Health Policy. 2022;126(5):373-81.

24. Booton RD, Macgregor L, Vass L, Looker KJ, Hyams C, Bright PD, et al. Estimating the COVID-19 epidemic trajectory and hospital capacity requirements in South West England: A mathematical modelling framework. BMJ Open. 2021;11(1).

25. Boussarsar M, Meddeb K, Toumi R, Ennouri E, Ayed S, Jarraya F, et al. Resource utilization and preparedness within the COVID-19 pandemic in Tunisian medical intensive care units: A nationwide retrospective multicentre observational study. Journal of Infection and Public Health. 2023;16(5):727-35.

26. Braeye T, Catteau L, Brondeel R, van Loenhout JAF, Proesmans K, Cornelissen L, et al. Vaccine effectiveness against transmission of alpha, delta and omicron SARS-COV-2-infection, Belgian contact tracing, 2021–2022. Vaccine. 2023;41(20):3292-300.

27. Canga A, Bidegain G. Modelling the Effect of the Interaction between Vaccination and Nonpharmaceutical Measures on COVID-19 Incidence. Global Health, Epidemiology and Genomics. 2022;2022.

28. Castagna F, Xue X, Saeed O, Kataria R, Puius YA, Patel SR, et al. Hospital bed occupancy rate is an independent risk factor for COVID-19 inpatient mortality: A pandemic epicentre cohort study. BMJ Open. 2022;12(2).

29. Cattaneo A, Vitali A, Mazzoleni M, Previdi F. An agent-based model to assess large-scale COVID-19 vaccination campaigns for the Italian territory: The case study of Lombardy region. Computer Methods and Programs in Biomedicine. 2022;224.

30. Chalk D, Robbins S, Kandasamy R, Rush K, Aggarwal A, Sullivan R, Chamberlain C. Modelling palliative and end-of-life resource requirements during COVID-19: Implications for quality care. BMJ Open. 2021;11(5).

31. Challen K, Bentley A, Bright J, Walter D. Clinical review: Mass casualty triage - Pandemic influenza and critical care. Critical Care. 2007;11(2).

32. Chappell L, Horby P, Lim W, The RECOVERY Collaborative Group. Dexamethasone in Hospitalized Patients with Covid-19. New England Journal of Medicine. 2020;384(8):693-704.

33. Chen C, Luo Q, Chong N, Westergaard S, Brantley E, Salsberg E, et al. Coronavirus Disease 2019 Planning and Response: A Tale of 2 Health Workforce Estimator Tools. Medical care. 2021;59:S420-S7.

34. Chhim S, Ku G, Mao S, Van De Put W, Van Damme W, Ir P, et al. Descriptive assessment of COVID-19 responses and lessons learnt in Cambodia, January 2020 to June 2022. BMJ Global Health. 2023;8(5).

35. Chomton M, Marsac L, Deho A, Maroni A, Geslain G, Frannais-Haverland K, et al. Transforming a paediatric ICU to an adult ICU for severe Covid-19: lessons learned. European Journal of Pediatrics. 2021;180(7):2319-23.

36. Chu C, Lee J, Choi DH, Youn SK, Lee JK. Sensitivity Analysis of the Parameters of Korea's Pandemic Influenza Preparedness Plan. Osong Public Health and Research Perspectives. 2011;2(3):210-5.

37. Chua BWB, Huynh VA, Lou J, Goh FT, Clapham H, Teerawattananon Y, Wee HL. Protocol for the economic evaluation of COVID-19 pandemic response policies. BMJ Open. 2021;11(9).

38. Considine J, Shaban RZ, Patrick J, Holzhauser K, Aitken P, Clark M, et al. Pandemic (H1N1) 2009 Influenza in Australia: Absenteeism and redeployment of emergency medicine and nursing staff. EMA - Emergency Medicine Australasia. 2011;23(5):615-23.

39. Cruz-Aponte M, McKiernan EC, Herrera-Valdez MA. Mitigating effects of vaccination on influenza outbreaks given constraints in stockpile size and daily administration capacity. BMC Infectious Diseases. 2011;11.

40. Cummings MJ, Baldwin MR, Abrams D, Jacobson SD, Meyer BJ, Balough EM, et al. Epidemiology, clinical course, and outcomes of critically ill adults with COVID-19 in New York City: a prospective cohort study. The Lancet. 2020;395(10239):1763-70.

41. Dadhwal K, Stonham R, Breen H, Poole S, Saeed K, Dushianthan A. Severe COVID-19 pneumonia in an intensive care setting and comparisons with historic severe viral pneumonia due to other viruses. Clinical Respiratory Journal. 2022;16(4):301-8.

42. Datta BK, Ansa BE, George V. An analytical model of population level chronic conditions and COVID-19 related hospitalization in the United States. BMC public health. 2022;22(1):208.

43. Davis EL, Lucas TCD, Borlase A, Pollington TM, Abbott S, Ayabina D, et al. Contact tracing is an imperfect tool for controlling COVID-19 transmission and relies on population adherence. Nature Communications. 2021;12(1).

44. de León UAP, Avila-Vales E, Huang K. Modeling the Transmission of the SARS-CoV-2 Delta Variant in a Partially Vaccinated Population. Viruses. 2022;14(1).

45. de Wolff T, Pflüger D, Rehme M, Heuer J, Bittner MI. Evaluation of pool-based testing approaches to enable population-wide screening for COVID-19. PLoS ONE. 2021;15(12 December).

46. Doron S, Monach PA, Brown CM, Branch-Elliman W. Improving COVID-19 Disease Severity Surveillance Measures: Statewide Implementation Experience. Annals of internal medicine. 2023.

47. Doyle A, Bonmarin I, Lévy-Bruhl D, Le Strat Y, Desenclos JC. Influenza pandemic preparedness in France: Modelling the impact of interventions. Journal of Epidemiology and Community Health. 2006;60(5):399-404.

48. Elhadi M, Alsoufi A, Abusalama A, Alkaseek A, Abdeewi S, Yahya M, et al. Epidemiology, outcomes, and utilization of intensive care unit resources for critically ill COVID-19 patients in Libya: A prospective multi-center cohort study. PLoS ONE. 2021;16(4 April).

49. Elhakim M, Tourab SB, Salem F, Van De Weerdt R. Epidemiology of the first and second waves of COVID-19 pandemic in Djibouti and the vaccination strategy developed for the response. BMJ Global Health. 2022;7.

50. Ercole A, Taylor BL, Rhodes A, Menon DK. Modelling the impact of an influenza A/H1N1 pandemic on critical care demand from early pathogenicity data: The case for sentinel reporting. Anaesthesia. 2009;64(9):937-41.

51. Eyre DW, Futschik M, Tunkel S, Wei J, Cole-Hamilton J, Saquib R, et al. Performance of antigen lateral flow devices in the UK during the alpha, delta, and omicron waves of the SARS-CoV-2 pandemic: a diagnostic and observational study. The Lancet Infectious Diseases. 2023.

52. Fort D, Seoane L, Unis GD, Price-Haywood EG. Locally informed modeling to predict hospital and intensive care unit capacity during the COVID-19 epidemic. Ochsner Journal. 2020;20(3):285-92.

53. Galante M, Garin O, Sicuri E, Cots F, García-Altés A, Ferrer M, et al. Health services utilization, work absenteeism and costs of pandemic influenza A (H1N1) 2009 in Spain: A multicenter-longitudinal study. PLoS ONE. 2012;7(2).

54. Gelfman LP, Moreno J, Frydman JL, Singer J, Houldsworth J, Cordon-Cardo C, et al. Characteristics Associated With Disparities Among Older Adults in Coronavirus Disease 2019 Outcomes in an Academic Health Care System. Medical care. 2022.

55. Giannakeas V, Bhatia D, Warkentin MT, Bogoch II, Stall NM. Estimating the Maximum Capacity of COVID-19 Cases Manageable per Day Given a Health Care System's Constrained Resources. Annals of internal medicine. 2020;173(5):407-10.

56. Godeaux O, Izurieta P, Madariaga M, Dramé M, Li P, Vaughn DW. Immunogenicity and safety of AS03<inf>A</inf>-adjuvanted H5N1 influenza vaccine prepared from bulk antigen after stockpiling for 4 years. Vaccine. 2015;33(18):2189-95.

57. Grasselli G, Zangrillo A, Zanella A, Antonelli M, Cabrini L, Castelli A, et al. Baseline characteristics and outcomes of 1591 patients infected with SARS-CoV-2 admitted to ICUs of the Lombardy Region, Italy. Jama. 2020;323(16):1574-81.

58. Haase N, Plovsing R, Christensen S, Poulsen LM, Brøchner AC, Rasmussen BS, et al. Changes over time in characteristics, resource use and outcomes among ICU patients with COVID-19—A nationwide, observational study in Denmark. Acta Anaesthesiologica Scandinavica. 2022;66(8):987-95.

59. Hara Y, Hagihara A, Ikematu H, Nobutomo K. Efficacy of influenza vaccine among elderly patients by physical activity status. Environmental Health and Preventive Medicine. 2002;7(5):183-8.

60. Hashikura M, Kizu J. Stockpile of personal protective equipment in hospital settings: Preparedness for influenza pandemics. American Journal of Infection Control. 2009;37(9):703-7.

61. Hayden E, Airoldi C, Scotti L, Bellan M, Sottosanti A, Bergamasco P, et al. Modelling hospital bed necessity for COVID-19 patients during the decline phase of the epidemic trajectory. Minerva Respiratory Medicine. 2021;60(2):29-35.

62. Hertzberg D, Renberg M, Nyman J, Bell M, Rimes Stigare C. Experiences of Renal Replacement Therapy Delivery in Swedish Intensive Care Units during the COVID-19 Pandemic. Blood Purification. 2021:1-6.

63. Ho PI, Liu W, Li TZR, Chan TC, Ku CC, Lien YH, et al. Taiwan's Response to Influenza: A Seroepidemiological Evaluation of Policies and Implications for Pandemic Preparedness. International Journal of Infectious Diseases. 2022;121:226-37.

64. Hohl CM, Rosychuk RJ, Hau JP, Hayward J, Landes M, Yan JW, et al. Treatments, resource utilization, and outcomes of COVID-19 patients presenting to emergency departments across pandemic waves: an observational study by the Canadian COVID-19 Emergency Department Rapid Response Network (CCEDRRN). Canadian Journal of Emergency Medicine. 2022;24(4):397-407.

65. Hong SA. Six Pivotal Lessons Learned in South Korea for Whole-of-Government Approach to Successful COVID-19 Vaccine Rollout in Planetary Health. OMICS A Journal of Integrative Biology. 2022;26(10):567-79.

66. Hsu L, Grüne B, Buess M, Joisten C, Klobucnik J, Nießen J, et al. Covid-19 breakthrough infections and transmission risk: Real-world data analyses from germany’s largest public health department (cologne). Vaccines. 2021;9(11).

67. Huang HC, Araz OM, Morton DP, Johnson GP, Damien P, Clements B, Meyers LA. Stockpiling ventilators for influenza pandemics. Emerging Infectious Diseases. 2017;23(6):914-21.

68. Iragorri N, Gómez-Restrepo C, Barrett K, Herrera S, Hurtado I, Khan Y, et al. COVID-19: Adaptation of a model to predicting healthcare resources needs in Valle del Cauca, Colombia. Colombia medica (Cali, Colombia). 2020;51(3):e204534.

69. Irvine N, Anderson G, Sinha C, McCabe H, van der Meer R. Collaborative critical care prediction and resource planning during the COVID-19 pandemic using computer simulation modelling: Future urgent planning lessons. Future Healthcare Journal. 2021;8(2):E317-E21.

70. Islam MS, Bhowmick DK, Parveen M, Kamal MM, Akhtaruzzaman AKM. Case fatality rate and survival functions of severe COVID-19 patients in intensive care unit of Bangabandhu Sheikh Mujib Medical University in Bangladesh: An observational study. Anaesthesia, Pain and Intensive Care. 2021;25(4):443-9.

71. Jost D, Derkenne C, Kedzierewicz R, Briche F, Frattini B, Bertho K, et al. The need to adapt the rescue chain for out-of-hospital cardiac arrest during the COVID-19 pandemic: Experience from the Paris Fire Brigade Basic Life Support and Advanced Life Support teams. Resuscitation. 2020;153:56-7.

72. Kafan S, Vajargah KT, Sheikhvatan M, Tabrizi G, Salimzadeh A, Montazeri M, et al. Predicting risk score for mechanical ventilation in hospitalized adult patients suffering from covid-19. Anesthesiology and Pain Medicine. 2021;11(2).

73. Kasaie P, Kelton WD. Resource allocation for controlling epidemics: Calibrating, analyzing, and optimizing an agent-based simulation. IIE Transactions on Healthcare Systems Engineering. 2013;3(2):94-109.

74. Kasturi SN, Park J, Wild D, Khan B, Haggstrom DA, Grannis S. Predicting COVID-19-related health care resource utilization across a statewide patient population: Model development study. Journal of Medical Internet Research. 2021;23(11).

75. Khawaja SN, Qadri HA, Yusuf MA. Cancer hospital stockpiles: Strategizing for an efficient and sufficient inventory list of essential items. JCO Global Oncology. 2021(7):1490-9.

76. Kriegova E, Fillerova R, Raska M, Manakova J, Dihel M, Janca O, et al. Excellent option for mass testing during the SARS-CoV-2 pandemic: painless self-collection and direct RT-qPCR. Virology Journal. 2021;18(1).

77. Krumkamp R, Kretzschmar M, Rudge JW, Ahmad A, Hanvoravongchai P, Westenhoefer J, et al. Health service resource needs for pandemic influenza in developing countries: A linked transmission dynamics, interventions and resource demand model. Epidemiology and Infection. 2011;139(1):59-67.

78. Laake JH, Buanes EA, Småstuen MC, Kvåle R, Olsen BF, Rustøen T, et al. Characteristics, management and survival of ICU patients with coronavirus disease-19 in Norway, March-June 2020. A prospective observational study. Acta Anaesthesiologica Scandinavica. 2021;65(5):618-28.

79. Lam SSW, Pourghaderi AR, Abdullah HR, Nguyen FNHL, Siddiqui FJ, Ansah JP, et al. An Agile Systems Modeling Framework for Bed Resource Planning During COVID-19 Pandemic in Singapore. Frontiers in public health. 2022;10:714092.

80. Lawandi A, Warner S, Sun J, Demirkale CY, Danner RL, Klompas M, et al. Suspected Severe Acute Respiratory Syndrome Coronavirus 2 (SARS-COV-2) Reinfections: Incidence, Predictors, and Healthcare Use Among Patients at 238 US Healthcare Facilities, 1 June 2020 to 28 February 2021. Clinical Infectious Diseases. 2022;74(8):1489-92.

81. Lee HY, Oh MN, Park YS, Chu C, Son TJ. Public Health Crisis Preparedness and Response in Korea. Osong Public Health and Research Perspectives. 2013;4(5):278-84.

82. Lee VJ, Chen MI. Effectiveness of neuraminidase inhibitors for preventing staff absenteeism during pandemic influenza. Emerging Infectious Diseases. 2007;13(3):449-57.

83. Lee BY, Bailey RR, Wiringa AE, Assi TM, Beigi RH. Antiviral medications for pregnant women for pandemic and seasonal influenza: An economic computer model. Obstetrics and Gynecology. 2009;114(5):971-80.

84. Lee BY, McGlone SM, Bailey RR, Wiringa AE, Zimmer SM, Smith KJ, Zimmerman RK. To test or to treat? an analysis of influenza testing and Antiviral treatment strategies using economic computer modeling. PLoS ONE. 2010;5(6).

85. Li Q, Huang Y. Optimizing global COVID-19 vaccine allocation: An agent-based computational model of 148 countries. PLoS Computational Biology. 2022;18(9).

86. Mahévas M, Tran V-T, Roumier M, Chabrol A, Paule R, Guillaud C, et al. Clinical efficacy of hydroxychloroquine in patients with covid-19 pneumonia who require oxygen: observational comparative study using routine care data. Bmj. 2020;369.

87. Marijon E, Karam N, Jost D, Perrot D, Frattini B, Derkenne C, et al. Out-of-hospital cardiac arrest during the COVID-19 pandemic in Paris, France: a population-based, observational study. The Lancet Public Health. 2020;5(8):e437-e43.

88. Mayorga L, García Samartino C, Flores G, Masuelli S, Sánchez MV, Mayorga LS, Sánchez CG. A modelling study highlights the power of detecting and isolating asymptomatic or very mildly affected individuals for COVID-19 epidemic management. BMC public health. 2020;20(1):1809.

89. McCabe R, Kont MD, Schmit N, Whittaker C, Løchen A, Baguelin M, et al. Modelling intensive care unit capacity under different epidemiological scenarios of the COVID-19 pandemic in three Western European countries. International journal of epidemiology. 2021.

90. Medema JK, Zoellner YF, Ryan J, Palache AM. Modeling pandemic preparedness scenarios: Health economic implications of enhanced pandemic vaccine supply. Virus Research. 2004;103(1-2):9-15.

91. Melman GJ, Parlikad AK, Cameron EAB. Balancing scarce hospital resources during the COVID-19 pandemic using discrete-event simulation. Health care management science. 2021.

92. Merler S, Ajelli M, Rizzo C. Age-prioritized use of antivirals during an influenza pandemic. BMC Infectious Diseases. 2009;9.

93. Methi F, Hernæs KH, Skyrud KD, Magnusson K. Pandemic trends in health care use: From the hospital bed to self-care with COVID-19. PLoS ONE. 2022;17(3 March).

94. Miller G, Randolph S, Patterson JE. Responding to simulated pandemic influenza in San Antonio, Texas. Infection Control and Hospital Epidemiology. 2008;29(4):320-6.

95. Milne G, Kelso J, Kelly H. Strategies for mitigating an influenza pandemic with pre-pandemic H5N1 vaccines. Journal of the Royal Society Interface. 2010;7(45):573-86.

96. Modisenyane M, Madikezela L, Mngemane S, Ramadan OP, Matlala M, McCarthy K, et al. COVID-19 response in South African communities: Screening, testing, tracing and movement modelling. South African Medical Journal. 2022;112(5b):366-70.

97. Moon RC, Brown H, Rosenthal N. Healthcare Resource Utilization of Patients With COVID-19 Visiting US Hospitals. Value in Health. 2022;25(5):751-60.

98. Nap RE, Andriessen MPHM, Meessen NEL, Dos Reis Miranda D, Van Der Werf TS. Pandemic influenza and excess intensive-care workload. Emerging Infectious Diseases. 2008;14(10):1518-25.

99. Nap RE, Andriessen MPHM, Meessen NEL, Van Der Werf TS. Pandemic influenza and hospital resources. Emerging Infectious Diseases. 2007;13(11):1714-9.

100. Nicolay N, Callaghan MA, Domegan LM, Oza AN, Marsh BJ, Flanagan PC, et al. Epidemiology, clinical characteristics and resource implications of pandemic (H1N1) 2009 in intensive care units in Ireland. Critical care and resuscitation : journal of the Australasian Academy of Critical Care Medicine. 2010;12(4):255-61.

101. Noreen N, Naveed I, Dil S, Niazi S, Saleem S, Mohiuddin N, et al. Trend Analysis of exponential increase of Covid-19 cases in Pakistan: An interpretation. Global Biosecurity. 2020;2(1).

102. Nuño M, Chowell G, Gumel AB. Assessing the role of basic control measures, antivirals and vaccine in curtailing pandemic influenza: Scenarios for the US, UK and the Netherlands. Journal of the Royal Society Interface. 2007;4(14):505-21.

103. Oakley C, Pascoe C, Balthazor D, Bennett D, Gautam N, Isaac J, et al. Assembly Line ICU: what the Long Shops taught us about managing surge capacity for COVID-19. BMJ open quality. 2020;9(4).

104. Opstelten W, Van Steenbergen JE, Van Essen GA, Van Der Sande MAB. The use of antiviral agents during an (impending) influenza pandemic

Het gebruik van antivirale middelen tijdens een (dreigende) influenzapandemie. Nederlands Tijdschrift voor Geneeskunde. 2007;151(18):1008-12.

105. Pastorino R, Villani L, La Milia DI, Ieraci R, Chini F, Volpe E, et al. Influenza and pneumococcal vaccinations are not associated to COVID-19 outcomes among patients admitted to a university hospital. Vaccine. 2021;39(26):3493-7.

106. Pham TM, Tahir H, van de Wijgert JHHM, Van der Roest BR, Ellerbroek P, Bonten MJM, et al. Interventions to control nosocomial transmission of SARS-CoV-2: a modelling study. BMC Medicine. 2021;19(1).

107. Philips S, Shi Y, Coopersmith CM, Samuels OB, Pimentel-Farias C, Mei Y, et al. Surge Capacity in the COVID-19 Era: a Natural Experiment of Neurocritical Care in General Critical Care. Neurocritical Care. 2023;38(2):320-5.

108. Phin NF, Rylands AJ, Allan J, Edwards C, Enstone JE, Nguyen-Van-Tam JS. Personal protective equipment in an influenza pandemic: a UK simulation exercise. Journal of Hospital Infection. 2009;71(1):15-21.

109. Prada SI, Garcia-Garcia MP, Guzman J. COVID-19 response in Colombia: Hits and misses. Health Policy and Technology. 2022;11(2).

110. Rainisch G, Jeon S, Pappas D, Spencer KD, Fischer LS, Adhikari BB, et al. Estimated COVID-19 Cases and Hospitalizations Averted by Case Investigation and Contact Tracing in the US. JAMA Network Open. 2022;5(3):E224042.

111. Ravikumar N, Nallasamy K, Bansal A, Angurana SK, Basavaraja GV, Sundaram M, et al. Novel Coronavirus 2019 (2019-nCoV) Infection: Part I - Preparedness and Management in the Pediatric Intensive Care Unit in Resource-limited Settings. Indian Pediatrics. 2020;57(4):324-34.

112. Rennert-May E, Crocker A, D’Souza AG, Zhang Z, Chew D, Beall R, et al. Healthcare utilization and adverse outcomes stratified by sex, age and long-term care residency using the Alberta COVID-19 Analytics and Research Database (ACARD): a population-based descriptive study. BMC Infectious Diseases. 2023;23(1).

113. Richardson S, Hirsch JS, Narasimhan M, Crawford JM, McGinn T, Davidson KW, et al. Presenting characteristics, comorbidities, and outcomes among 5700 patients hospitalized with COVID-19 in the New York City area. Jama. 2020;323(20):2052-9.

114. Ritter M, Ott DVM, Paul F, Haynes JD, Ritter K. COVID-19: a simple statistical model for predicting intensive care unit load in exponential phases of the disease. Scientific reports. 2021;11(1):5018.

115. Rosenberg ES, Dufort EM, Udo T, Wilberschied LA, Kumar J, Tesoriero J, et al. Association of treatment with hydroxychloroquine or azithromycin with in-hospital mortality in patients with COVID-19 in New York State. Jama. 2020;323(24):2493-502.

116. Ruggeri M, Signorini A, Caravaggio S, Rua J, Luís N, Braz S, Aragão F. Estimation Model for Healthcare Costs and Intensive Care Units Access for Covid-19 Patients and Evaluation of the Effects of Remdesivir in the Portuguese Context: Hypothetical Study. Clinical Drug Investigation. 2022;42(4):345-54.

117. Ruggeri M, Signorini A, Caravaggio S, Alraddadi B, Alali A, Jarrett J, et al. Modeling the Potential Impact of Remdesivir Treatment for Hospitalized Patients with COVID-19 in Saudi Arabia on Healthcare Resource Use and Direct Hospital Costs: A Hypothetical Study. Clinical Drug Investigation. 2022;42(8):669-78.

118. Sander B, Nizam A, Garrison Jr LP, Postma MJ, Halloran ME, Longini Jr IM. Economic evaluation of influenza pandemic mitigation strategies in the United States using a stochastic microsimulation transmission model. Value in Health. 2009;12(2):226-33.

119. Saunders-Hastings P, Hayes BQ, Smith R, Krewski D. National assessment of Canadian pandemic preparedness: Employing InFluNet to identify high-risk areas for inter-wave vaccine distribution. Infectious Disease Modelling. 2017;2(3):341-52.

120. Scott A, Chambers R, Reimbaeva M, Atwell J, Baillon-Plot N, Draica F, Tarallo M. Real-world retrospective analysis of patient characteristics, healthcare resource utilization, costs, and treatment patterns among unvaccinated adults with COVID-19 diagnosed in outpatient settings in the United States. Journal of Medical Economics. 2022;25(1):287-98.

121. Sharma A, Oda G, Icardi M, Mole L, Holodniy M. Implementation of large-scale laboratory-based detection of COVID-19 in the Veterans Health Administration, March 2020 – February 2021. Diagnostic Microbiology and Infectious Disease. 2022;102(3).

122. Smetanin P, Stiff D, Kumar A, Kobak P, Zarychanski R, Simonsen N, Plummer F. Potential intensive care unit ventilator demand/capacity mismatch due to novel swine-origin H1N1 in Canada. Canadian Journal of Infectious Diseases and Medical Microbiology. 2009;20(4):e115-e23.

123. Solanki G, Wilkinson T, Bansal S, Shiba J, Manda S, Doherty T. COVID-19 hospitalization and mortality and hospitalization-related utilization and expenditure: Analysis of a South African private health insured population. PLoS ONE. 2022;17(5 May).

124. Sritipsukho P, Khawcharoenporn T, Siribumrungwong B, Damronglerd P, Suwantarat N, Satdhabudha A, et al. Comparing real-life effectiveness of various COVID-19 vaccine regimens during the delta variant-dominant pandemic: a test-negative case-control study. Emerging Microbes and Infections. 2022;11(1):585-92.

125. Stanislawski N, Lange F, Fahnemann C, Riggers C, Wahalla MN, Porr M, et al. Mobile SARS‑CoV‑2 screening facilities for rapid deployment and university-based diagnostic laboratory. Engineering in Life Sciences. 2023;23(2).

126. Stein ML, Rudge JW, Coker R, van der Weijden C, Krumkamp R, Hanvoravongchai P, et al. Development of a resource modelling tool to support decision makers in pandemic influenza preparedness: The AsiaFluCap Simulator. BMC public health. 2012;12:870.

127. Stepanova M, Lam B, Younossi E, Felix S, Ziayee M, Price J, et al. The impact of variants and vaccination on the mortality and resource utilization of hospitalized patients with COVID-19. BMC Infectious Diseases. 2022;22(1).

128. Stiff D, Kumar A, Kissoon N, Fowler R, Jouvet P, Skippen P, et al. Potential pediatric intensive care unit demand/capacity mismatch due to novel pH1N1 in Canada. Pediatric Critical Care Medicine. 2011;12(2):e51-e7.

129. Subiros M, De Latour CR, Parenton F, Soulaimana I, Hassani Y, Blondé R, et al. Epidemiological profile of COVID-19 in the French overseas department Mayotte, 2020 to 2021. Eurosurveillance. 2022;27(34).

130. Suetens C, Kinross P, Berciano PG, Nebreda VA, Hassan E, Calba C, et al. Increasing risk of breakthrough COVID-19 in outbreaks with high attack rates in European long-term care facilities, July to October 2021. Eurosurveillance. 2021;26(49).

131. Swaminathan A, Martin R, Gamon S, Aboltins C, Athan E, Braitberg G, et al. Personal protective equipment and antiviral drug use during hospitalization for suspected avian or pandemic influenza. Emerging Infectious Diseases. 2007;13(10):1541-7.

132. Taboe HB, Asare-Baah M, Iboi EA, Ngonghala CN. Critical assessment of the impact of vaccine-type and immunity on the burden of COVID-19. Mathematical Biosciences. 2023;360.

133. Ten Eyck RP. Ability of regional hospitals to meet projected avian flu pandemic surge capacity requirements. Prehospital and disaster medicine : the official journal of the National Association of EMS Physicians and the World Association for Emergency and Disaster Medicine in association with the Acute Care Foundation. 2008;23(2):103-12.

134. Tran Kiem C, Bosetti P, Paireau J, Crépey P, Salje H, Lefrancq N, et al. SARS-CoV-2 transmission across age groups in France and implications for control. Nature Communications. 2021;12(1).

135. Trentini F, Marziano V, Guzzetta G, Tirani M, Cereda D, Poletti P, et al. Pressure on the Health-Care System and Intensive Care Utilization During the COVID-19 Outbreak in the Lombardy Region of Italy: A Retrospective Observational Study in 43,538 Hospitalized Patients. American Journal of Epidemiology. 2022;191(1):137-46.

136. Tuite AR, Fisman DN, Kwong JC, Greer AL. Optimal pandemic influenza vaccine allocation strategies for the Canadian population. PLoS ONE. 2010;5(5).

137. Van Genugten MLL, Heijnen MLA. The expected number of hospitalisations and beds needed due to pandemic influenza on a regional level in the Netherlands. Virus Research. 2004;103(1-2):17-23.

138. Verma VR, Saini A, Gandhi S, Dash U, Koya SF. Capacity-need gap in hospital resources for varying mitigation and containment strategies in India in the face of COVID-19 pandemic. Infectious Disease Modelling. 2020;5:608-21.

139. Vidal-Cortés P, Martín MC, Díaz E, Bodí M, Igeño JC, Garnacho-Montero J. Impact of one year of pandemic on Spanish Intensive Care Units

Impacto de un año de pandemia en las Unidades de Cuidados Intensivos de España. Revista Espanola de Quimioterapia. 2022;35(4):392-400.

140. Wang Y, Zhang D, Du G, Du R, Zhao J, Jin Y, et al. Remdesivir in adults with severe COVID-19: a randomised, double-blind, placebo-controlled, multicentre trial. The lancet. 2020;395(10236):1569-78.

141. Webb E, Winkelmann J, Scarpetti G, Behmane D, Habicht T, Kahur K, et al. Lessons learned from the Baltic countries’ response to the first wave of COVID-19. Health Policy. 2022;126(5):438-45.

142. Weissman GE, Crane-Droesch A, Chivers C, Luong T, Hanish A, Levy MZ, et al. Locally Informed Simulation to Predict Hospital Capacity Needs During the COVID-19 Pandemic. Annals of internal medicine. 2020;173(1):21-8.

143. Wood J, McCaw J, Becker N, Nolan T, MacIntyre CR. Optimal dosing and dynamic distribution of vaccines in an influenza pandemic. American Journal of Epidemiology. 2009;169(12):1517-24.

144. Wood RM, McWilliams CJ, Thomas MJ, Bourdeaux CP, Vasilakis C. COVID-19 scenario modelling for the mitigation of capacity-dependent deaths in intensive care. Health care management science. 2020;23(3):315-24.

145. Zhang L, Chai L, Zhao Y, Wang L, Sun W, Lu L, et al. Burnout in nurses during the COVID-19 pandemic in China: New challenges for public health. Bioscience trends. 2021;15(2):129-31.

146. Zhang X, Meltzer MI, Wortley PM. FluSurge - A tool to estimate demand for hospital services during the next pandemic influenza. Medical Decision Making. 2006;26(6):617-23.

147. Zirbes J, Sterr CM, Keller C, Engenhart-Cabillic R, Nonnenmacher-Winter C, Günther F. Efficiency analysis of rapid antigen test based SARS-CoV-2 in hospital contact tracing and screening regime: test characteristics and cost effectiveness. Diagnostic Microbiology and Infectious Disease. 2023;106(4).
